# The incredible bulk: Human cytomegalovirus tegument architectures uncovered by AI-empowered cryo-EM

**DOI:** 10.1126/sciadv.adj1640

**Published:** 2024-02-23

**Authors:** Jonathan Jih, Yun-Tao Liu, Wei Liu, Z. Hong Zhou

**Affiliations:** ^1^ Molecular Biology Institute, University of California, Los Angeles (UCLA), Los Angeles, CA 90095, USA.; ^2^ California NanoSystems Institute, University of California, Los Angeles (UCLA), Los Angeles, CA 90095, USA.; ^3^ Department of Microbiology, Immunology, and Molecular Genetics, University of California, Los Angeles (UCLA), Los Angeles, CA 90095, USA.

## Abstract

The compartmentalization of eukaryotic cells presents considerable challenges to the herpesvirus life cycle. The herpesvirus tegument, a bulky proteinaceous aggregate sandwiched between herpesviruses’ capsid and envelope, is uniquely evolved to address these challenges, yet tegument structure and organization remain poorly characterized. We use deep-learning–enhanced cryogenic electron microscopy to investigate the tegument of human cytomegalovirus virions and noninfectious enveloped particles (NIEPs; a genome packaging-aborted state), revealing a portal-biased tegumentation scheme. We resolve atomic structures of portal vertex-associated tegument (PVAT) and identify multiple configurations of PVAT arising from layered reorganization of pUL77, pUL48 (large tegument protein), and pUL47 (inner tegument protein) assemblies. Analyses show that pUL77 seals the last-packaged viral genome end through electrostatic interactions, pUL77 and pUL48 harbor a head–linker–capsid-binding motif conducive to PVAT reconfiguration, and pUL47/48 dimers form 45-nm-long filaments extending from the portal vertex. These results provide a structural framework for understanding how herpesvirus tegument facilitates and evolves during processes spanning viral genome packaging to delivery.

## Teaser

Herpesviruses’ shape-shifting tegument betrays a structure well-adapted to facilitate viral propagation in a eukaryotic cell.

## INTRODUCTION

Human cytomegalovirus (HCMV) of the Herpesviridae β-herpesvirus subfamily is the leading infectious cause of birth defects (*1*, *2*) and major etiologic agent of high-mortality complications in the immunocompromised (*3*, *4*). Even in the mature and healthy, HCMV has increasingly been linked with high-burden chronic diseases of aging, no doubt related to HCMV’s exceptional ability among human herpesviruses to infect a remarkable range of cell types (*5*–*8*). While herpesviruses share similarities with their bacteriophage cousins, eukaryotic cells’ compartmentalized structure vastly increases the complexity of herpesvirus reproduction. Thus, with no equivalent in phages, the herpesvirus tegument is a unique adaptation shared by all three herpesvirus subfamilies that is the chief means of interacting with and navigating the intracellular landscape of their eukaryotic hosts (*9*, *10*).

Composed of a diverse array of proteins, the tegument is an amorphous aggregate sandwiched between herpesviruses’ icosahedral capsid and outer envelope (*11*). While tegument proteins in general are well-differentiated to carry out highly host-specific viral functions, tegument proteins related to the capsid vertex-specific component (CVSC) have in recent years been identified as key players mediating critical functions universal to all herpesviruses (*11*, *12*). These include in HCMV the CVSC proteins pUL77 [pUL25 in herpes simplex virus 1 (HSV-1)] and pUL48 (HSV-1 pUL36; also called large tegument protein), as well as pUL48’s non–CVSC-binding partner, pUL47 (HSV-1 pUL37; also called inner tegument protein). Tremendous biochemical and genetic efforts have implicated pUL77 and its homologs in capsid genome retention after packaging (*13*–*15*) and egress from the host nucleus (*16*), where nascent herpesvirus capsids are assembled. Meanwhile pUL48 and its homologs, as the largest among more than 100 proteins encoded by the herpes genome, are the Swiss Army knives of herpesviruses, which together with pUL47 and homologs facilitate evasion of innate cellular immunity (*17*, *18*), both kinesin- and dynein-mediated intracellular capsid transport (even assimilating cellular kinesin into mature virions) (*19*–*24*), acquisition of envelope-associated tegument and glycoproteins (*11*, *25*, *26*), nuclear pore complex (NPC) docking as a precursor to genome uncoating (*27*, *28*), and more (*9*, *11*, *29*–*31*). Amid this wealth of function, little is known of how these tegument components organize in a native, biologically relevant state. Although past structural studies have yielded some tegument crystal structures of select domains and fragments (*32*–*37*), in situ three-dimensional (3D) atomic characterization of tegument has lagged far behind that of its highly ordered capsid foundation (*38*–*46*). Key barriers driving this gap include tegument’s pleomorphic nature, frustrating the use of averaging for resolving high-resolution features; the sheer size of tegumented herpes virions (often >200 nm), posing technical challenges for microscopy and computation (*38*); and tegument’s lack of a functional analog in phages.

To lessen this gap and reveal the mechanistic details of tegument’s rich function, here, we deploy neural network–enhanced cryogenic electron tomography (cryo-ET) and single-particle cryogenic electron microscopy (cryo-EM) with symmetry relaxation to resolve tegument structures of HCMV virions and NIEPs (a genome packaging-aborted state). We find tegument clustered above the capsid’s unique DNA-translocating portal vertex and identify multiple configurations of portal vertex-associated tegument (PVAT) in both virion and NIEP populations, reflecting fully and partially tegumented states. Our atomic models of layered assemblies of pUL77, pUL47, and pUL48 evidence substantial PVAT rearrangement that delineate pleomorphic tegument’s large conformational space. This may be a critical feature necessary for tegument to orchestrate processes spanning viral genome packaging to delivery.

## RESULTS

### Deep learning enables direct observation of portal-referenced tegument distribution in HCMV enveloped particles

Efforts to directly interpret the tegument compartment of HCMV virions by cryo-ET have been frustrated by intrinsic missing-wedge artifacts (*47*) that obscure structural detail in the *x-z* and *y-z* planes of reconstructed tomograms. That the herpesvirus tegument is pleomorphic in nature precludes use of subtomogram averaging (STA) (*48*), a common strategy of averaging like-features to circumvent this problem and amplify otherwise low contrast features also inherent to cryo-ET. To overcome these limitations, we deployed IsoNet (*49*), a deep learning–based algorithm developed in our group to recover and correct for missing-wedge artifacts in tomographic data. For example, application of IsoNet to a previous cryo-ET dataset (*50*) showed markedly improved contrast and capsid features enabling direct structure interpretation from tomograms without the need for STA (fig. S1, A and B).

**Fig. 1.**
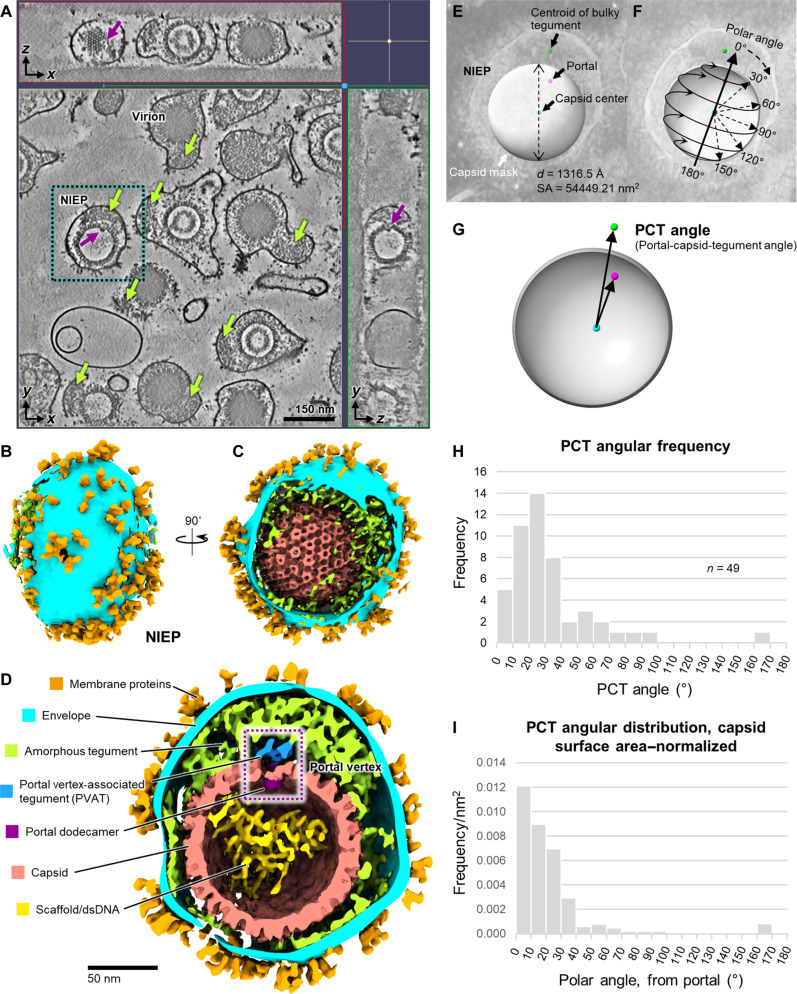
IsoNet-enhanced cryo-ET shows portal-referenced tegument in HCMV enveloped particles. (**A**) 2D slices of a reconstructed tomogram with missing-wedge correction by IsoNet (*49*) show HCMV virions and NIEPs with asymmetrically distributed tegument compartments (green arrows). Some NIEP capsids contain a spherical scaffold core, whereas others contain scattered density likely arising from broken scaffold proteins and/or incompletely packaged dsDNA genome. Portal dodecamer (purple arrow) is clearly visible in a NIEP. (**B**) NIEP boxed in (A) was computationally extracted and segmented. (**C**) Capsomer features are visible underneath the envelope. (**D**) Clipped view of (C) shows portal dodecamer and PVAT. (**E**) Mask diameter, *d*, is used to approximate the capsid’s surface area (SA). Coordinates of the portal, capsid center, and bulky tegument centroid are recorded using magenta, cyan, and lime green 3D markers, respectively. (**F**) Capsid polar angles are defined with respect to the portal vertex, taken to be the “north pole,” or 0°. The portal-opposite vertex, or the capsid’s “south pole” is 180°. (**G**) The PCT angle is defined as the angle between two vectors connecting capsid center to portal (cyan to magenta), and capsid center to the bulky tegument centroid (cyan to lime green). (**H**) Histogram showing measured PCT angular frequency sampled from 49 HCMV particles where portal complexes could be reasonably identified. (**I**) Histogram showing PCT angular distribution normalized to capsid surface area, revealing a tendency of tegument to localize above the portal vertex.

IsoNet-enhanced tomograms of HCMV enveloped particles reveal a mixed population of virions, distinguished by a dense core of packaged double-stranded DNA (dsDNA), and NIEPs, aberrant viral particles which lack a full complement of packaged genome within their capsid (Fig. 1A). Individual capsomers, hexon and penton channels, and envelope proteins are clearly visible in tomographic cross sections. We took special note of an asymmetric distribution of tegument between the capsid and viral envelope in >90% of our enveloped particles, consistent with previous observations of HSV-1 extracellular virions (*51*–*53*). These bulky and at times prominent distributions of tegument extend between 40 and 100 nm from the capsid to the viral envelope and rarely span an arc greater than 110° about the capsid surface (Fig. 1A and fig. S1, C to N).

As the herpesvirus dodecameric portal complex is situated on the interior surface of capsids, portal complex density is unfortunately obscured in many virions due to their dense, negatively charged genome core (*42*–*44*, *46*). In contrast, the lack of such a core in NIEPs permits positive identification of the portal complex and thereby the unique portal vertex on the icosahedral capsid. We observed that asymmetric tegument tends to aggregate above the portal vertex (Fig. 1A and fig. S1, C to N). We subsequently performed 3D segmentation of a NIEP, revealing capsid, portal, and tegument spatial relationships in greater clarity (Fig. 1, B to D). This permitted calculation of a “portal-capsid-tegument angle” (PCT angle), which we used as a proxy to quantify the angular distribution of tegument relative to the portal vertex (Fig. 1, E to G). Our analyses across 49 HCMV particles in which portal vertices could be identified confirmed correlation of bulky tegument distribution to the portal vertex, with most particles exhibiting asymmetric tegumentation centered within a 40° polar arc from the portal vertex (Fig. 1, H and I). Last, our segmentation revealed a small clustering of tegument density within the largely amorphous tegument compartment that was closely associated with the portal vertex. We recognized this feature to be consistent in both size and shape with prior cryo-EM descriptions of a PVAT structure (Fig. 1D) (*41*–*43*, *54*).

### Cryo-EM with sequential classification reveals multiple PVAT states but conserved global tegumentation

To further investigate PVAT structure, we performed single-particle cryo-EM of enveloped HCMV particles. Informed by our cryo-ET data of PVAT’s general location above the portal vertex, we targeted this region using symmetry-relaxed sequential subparticle classification, a cryo-EM data processing workflow we have previously implemented to resolve nested symmetric and asymmetric elements in icosahedral viral capsids (*42*, *43*). Owing to PVAT’s high radial distance from the capsid center and possible heterogeneity in its binding, we sought to maximize the number of particles (and thus possible signal) in our workflow input. Briefly, we classified 303,743 viral particles, of which 244,813 were virions and 58,930 were NIEPs. For both virion and NIEP populations, we separately extracted subparticles of capsid vertices and then used 3D classification to identify the unique portal vertex. We then performed localized 3D classification to resolve unique configurations of PVAT above the portal vertex (fig. S2). Our workflow netted subparticle reconstructions of two configurations of PVAT in virions [VC1 (virion configuration 1) and VC2 portal vertices at 3.50- and 3.27-Å resolution, respectively] and three configurations in NIEPs [NC1 (NIEP configuration 1), NC2, and NC2-inv (NIEP configuration 2, inverted) portal vertices at 4.26-, 4.29-, and 4.01-Å resolution, respectively] (figs. S3 and S4 and table S1). These results evidence that PVAT is not an unvarying static structure but exists in multiply configured states.

**Fig. 2.**
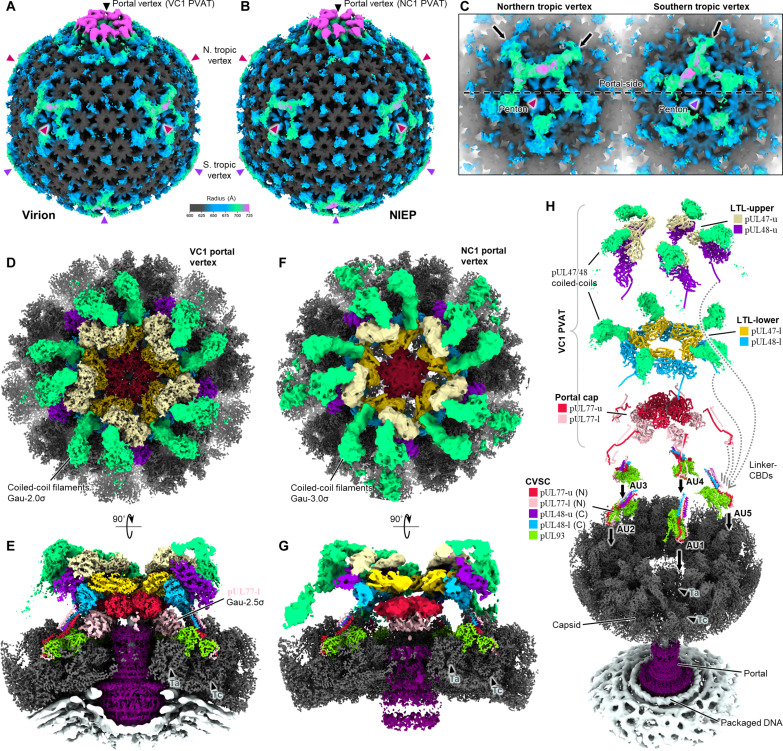
Cryo-EM reveals fully-tegumented PVAT structure in virions and NIEPs. (**A** and **B**) Global portal vertex-resolved reconstructions of VC1 virion (A) and NC1 NIEP (B) capsid. (**C**) Tropic penton vertices show preferred tegumentation at portal-proximal CVSC-binding-registers (black arrows). Dashed line is an equatorial drawn for reference. (**D** to **G**) Subparticle reconstructions of VC1 PVAT [(D) and (E)] and NC1 PVAT [(F) and (G)] segmented and colored as in (H). Contours chosen to best display overall structure features. (**H**) Exploded view showing components and layered organization of VC1-decorated portal vertex. AU1 to AU5 denotes asymmetric units 1 to 5.

We additionally back-extracted PVAT subparticle orientations to generate global portal-resolved capsid reconstructions for each unique virion and NIEP PVAT population (fig. S5 and table S1). When processed to emphasize low resolution features, these global capsid reconstructions reveal not just PVAT density crowning the portal vertex but also penton vertices ringed with globular tegument densities (Fig. 2, A and B, and fig. S5, A to D). Notably, across all PVAT types and in both virion and NIEP reconstructions, penton-associated tegument densities demonstrate biased occupancy toward the more portal-proximal CVSC-binding-registers of penton vertices [there are five possible CVSC-binding-registers per vertex (*43*, *46*)]. This is seen in the relative strength of tegument density (stronger density indicates higher occupancy) in the two prominently portal-proximal registers of northern tropic penton vertices, versus the more portal-distal registers (Fig. 2C, left; black arrows denote CVSC-binding-registers with high tegument occupancy). A similar phenomenon is observed in the single prominent portal-proximal register of the southern penton vertex (Fig. 2C, right). That these global reconstructions and the features they contain represent an average of many individual particles is both consistent with and further supports our cryo-ET data that suggest an asymmetric, portal-referenced scheme of tegumentation in mature viral particles.

### VC1 and NC1 PVAT structures represent fully tegumented portal vertices

We noticed similarities between two PVAT configurations, one virion and one NIEP. These we term "virion configuration 1" (VC1) and “NIEP configuration 1” (NC1) (fig. S4). Comparing subparticle reconstructions of VC1 and NC1 portal vertex, we observe analogous PVAT architecture characterized by three distinct layers of tegument protein that stack successively atop the portal complex and its integral DNA translocation channel (cf. Fig. 2, D to G, and movies S1 and S2).

The resolution of our VC1 portal vertex reconstruction was sufficient to identify components of and build atomic models for most of the visible tegument structure, which revealed that the three tegument layers are each a pentamer of dimers (Fig. 2H). An innermost layer consisting of five sets of pUL77 dimers constitutes a portal cap directly above the portal complex. Above the portal cap lies two large tegument layers (LTLs), each consisting of five subunits of pUL48 (large tegument protein) dimerized with a subunit of pUL47 (inner tegument protein). Together, VC1/NC1 PVAT comprises 10 copies of pUL77, 10 copies of pUL48, and 10 pUL48-corresponding copies of pUL47.

All three tegument layers anchor to the capsid via long flexible linkers connected to helix-rich capsid-binding domains (CBDs). We term these structural motifs “linker–CBDs,” which fulfill their tegument-anchoring roles by way of binding CVSC protein pUL93 (Fig. 2H, dotted arrows). As previously documented, five CVSCs decorate the five Ta-Tc triplex registers surrounding a herpesvirus portal vertex (*42*, *43*, *46*). Here, each CVSC consists of one pUL93 (HSV-1 pUL17’s homolog) that undergirds a helix bundle comprising two copies of pUL77 N terminus CBD (pUL25 in HSV-1) and two copies of pUL48 C terminus CBD (pUL36 in HSV-1). Hence, our VC1/NC1 PVAT structure accounts for the tegument proteins of all five CVSC asymmetric units encircling a portal vertex. We thus characterize VC1/NC1-decorated portal vertices as a fully tegumented state in which the entire complement of CVSC tegument proteins and pUL47 exhibit stable and orderly binding.

### Electrostatic interactions characterize structure and function of pUL77 portal cap

**Fig. 3.**
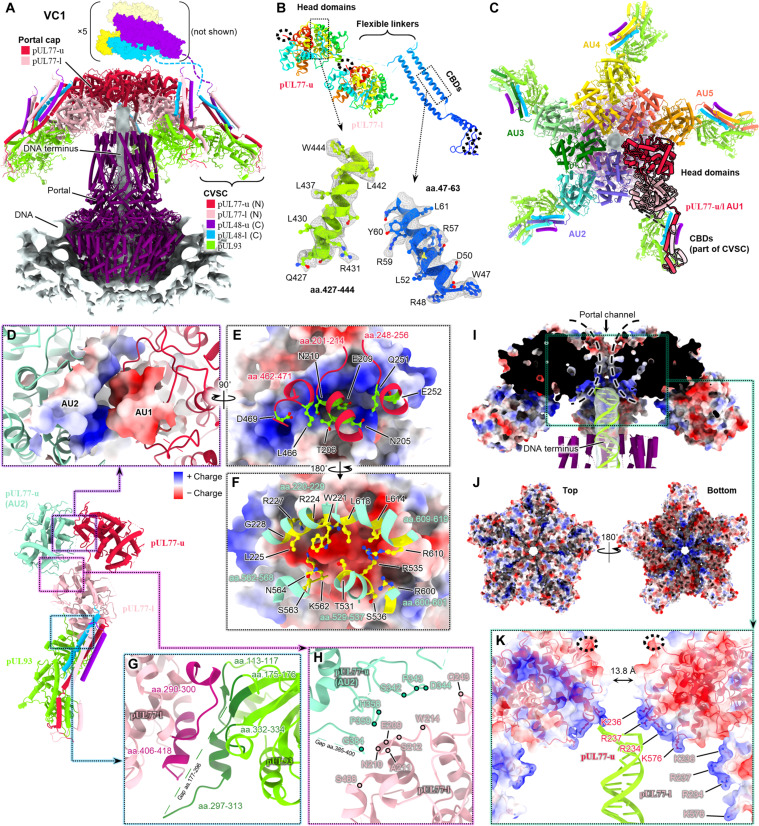
Electrostatic interactions characterize structure and function of pUL77 portal cap. (**A**) Overview of VC1 portal vertex shows two-layered pUL77 portal cap docked above pUL93 and portal channel. Models shown in cylinder-stub [portal model PDB 7ETM (*46*)]. (**B**) Atomic models of pUL77-u/l and local density fit. Rainbow coloring N to C (red to blue). Dashed circles denote termini. (**C**) Top view of portal cap, colored to demarcate asymmetric unit (AU) contributions. Five pUL77-u copies comprise the upper pentamer. (**D**) Adjacent pUL77-u pentamer subunits interlock via a positively charged “groove” (blue surface) and negatively charged “ridge” (red surface). (**E**) Ridge-forming residues of AU1 pUL77-u shown in lime green. (**F**) Groove-forming residues of AU2 pUL77-u shown in yellow. (**G**) Interfacing residue stretches between pUL77-l and pUL93 colored in maroon and dark green. (**H**) Interacting residues between AU2 pUL77-u and AU1 pUL77-l denoted by colored dots. (**I**) Clipped view of pUL77 portal cap rendered as electrostatic surface reveals a positively charged inverted “funnel” that retains DNA terminus’s rod-like density, shown fitted with ideal B-form DNA. (**J**) Top/bottom views of portal cap show the DNA-interfacing funnel is more positively charged (bottom view). (**K**) Lysine and arginine residues from pUL77-u/l line the funnel. Dashed circles denote exposed pUL77 C termini. aa., amino acids.

The 10 copies of pUL77 that cap VC1/NC1 portal complexes form a distinctly pentameric upper layer and a loosely aggregated lower layer (Fig. 2, E and G). In our VC1 structure (containing packaged viral DNA), the last-packaged genome terminus held within the portal’s DNA translocation channel contacts the underside of the upper pentamer layer (Fig. 3A). This is consistent with our work on HSV-1 and KSHV (Kaposi’s sarcoma-associated herpesvirus, a γ-herpesvirus) portal vertices, where we observed analogous structures of genome terminus capped within the DNA translocation channel by a low-resolution globular density (*42*, *43*). Owing to its apparent form, we had termed this globular density the “portal cap” and tentatively assigned its identity to the genome packaging-related HSV-1 pUL25 and its KSHV homolog pORF19, respectively. The higher resolution structure of HCMV’s equivalent here confirms the portal cap as being composed of pUL25 and pORF19’s HCMV homolog, pUL77.

Of full-length pUL77’s 642 amino acids, residues 1 to 180 form pUL77’s linker–CBD, whereas residues 181 to 642 form the C-terminal head domain that makes up the portal cap structure proper. Within the linker–CBD, residues 1 to 92 belong to the actual CBD that binds pUL93 and contributes to the CVSC helix bundle, whereas residues 93 to 180 form pUL77’s 88-residue-long flexible linker (largely disordered and unmodeled) (Fig. 3B). Our VC1 structure reveals that each CVSC asymmetric unit contributes an “upper” and “lower” copy of pUL77 to the portal cap, corresponding to the upper and lower pUL77 layers we observe (Fig. 3C and fig. S6, A to C). We refer to these copies as pUL77-u and pUL77-l, respectively.

The five pUL77-u copies that make up VC1 portal cap’s pentameric upper layer are relatively well-resolved and show strong density, indicating a stable well-ordered structure. Rendering pUL77-u by electrostatic surface potential reveals a prominent positively charged “groove” and negatively charged “ridge” on opposing faces of the head domain (Fig. 3D). Within the pentamer, pUL77-u subunits are oriented such that the positively charged groove of a subunit interfaces with the negatively charged ridge of an adjacent subunit (Fig. 3, E and F). Together, five pairs of charged grooves and ridges interlock the five pUL77-u pentamer subunits. We posit that this dual charge-steric complementarity imparts the structural integrity central to the portal cap’s ability to retain highly pressurized viral genome (*14*, *55*). Indeed, pentamerization of pUL77-u remains sufficiently robust to be observed even in the absence of packaged genome in NIEPs (Fig. 2G and figs. S4 and S6D).

In contrast, the five pUL77-l subunits of the lower layer are visibly less structured, exhibiting weak density in our VC1 reconstruction and being outright smeared in NC1 (cf. Fig. 2, E and G). While we thus were unable to natively model pUL77-l’s head domain, secondary structure elements were sufficiently resolved in our VC1 map to dock in an atomic model of pUL77-u head domain (fig. S6C). The resulting fit shows a nearly identical core head domain fold between pUL77-l and pUL77-u. Although lacking the complementarity of pUL77-u interactions, pUL77-l nonetheless assumes a fixed orientation in VC1 portal vertices, wedging between pUL93 and an adjacent CVSC’s pUL77-u (Fig. 3, G and H) (pUL77-u and pUL77-l dimerization is therefore interasymmetric unit). The specificity of this orientation is starkly illustrated in an electrostatic rendering of the complete portal cap structure—all 10 pUL77 subunits are arranged as to present a positively charged face toward the DNA translocation channel (Fig. 3, I and J). The end result is an inverted funnel lined with (positively-charged) arginine and lysine residues—namely, R234, K236, R237, and K576 from both pUL77-u and pUL77-l—that capture the genome terminus, presumably through interactions with dsDNA’s negatively charged backbone (Fig. 3K). By extension, we suspect that smearing of pUL77-l subunits in our NC1 map (Fig. 3G and fig. S6D) can be attributed to NC1’s lack of a captive genome terminus, thereby sterically permitting pUL77-l subunits to assume any which orientation. These results are consistent with both biochemical data (*14*, *15*), and our previous speculation (*42*, *43*) of HSV-1 and KSHV’s pUL77 homologs’ role as a portal cap in the context of genome retention, as well as recent speculation that electrostatic interactions in portal vertex-associated proteins are modulators of genome association (*33*, *56*).

### pUL48 C-terminal head dimerizes with pUL47 N-terminal fragment

**Fig. 4.**
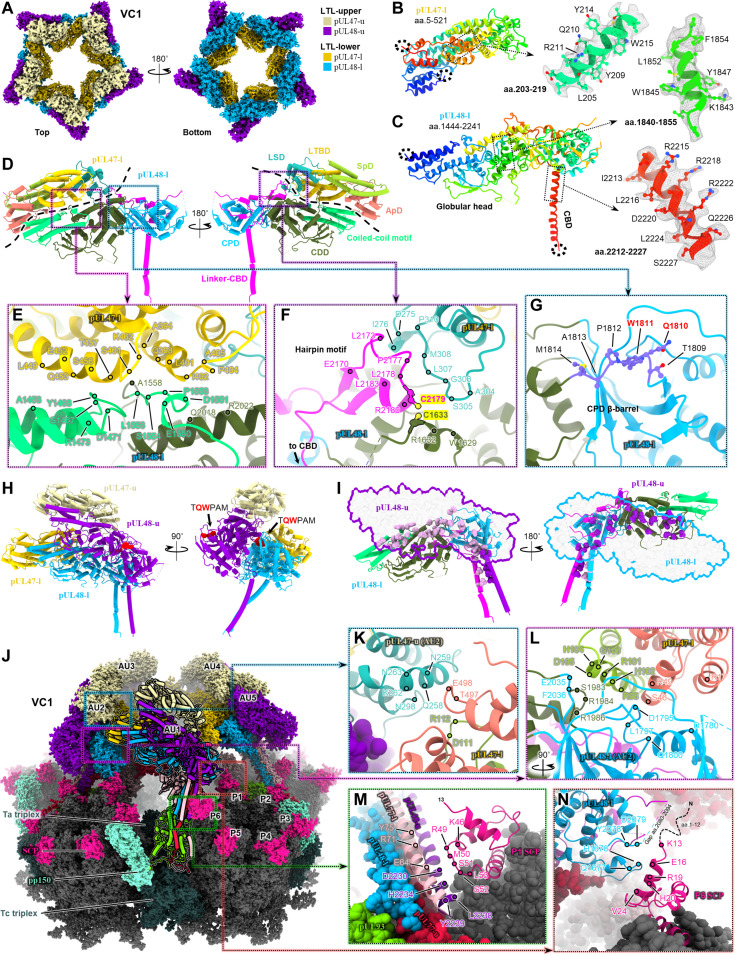
pUL47/48 dimerization underlies architecture of VC1/NC1 large tegument layers. (**A**) Top/bottom views of VC1 LTLs. (**B** and **C**) Atomic models of pUL47-l (B) and pUL48-l (C) and respective local density fit. Rainbow coloring N to C (red to blue). Dashed circles denote termini. (**D**) Cylinder-stub models of pUL47/48-l dimer colored by domain. Dashed lines demarcate dimer interface. pUL47 domains: ApD, apical domain; SpD, spine domain; LTBD, large-tegument–binding domain; LSD, linker-stabilizing domain. pUL48 domains: coiled-coil motif; CDD, capsid-distal domain; CPD, capsid-proximal domain; linker–CBD, linker–capsid-binding domain. (**E**) Primary subunit interface of pUL47/48 dimer. Interacting residues denoted by colored dots. (**F**) The linker of pUL48-l’s linker–CBD (magenta) forms an additional interface with pUL47-l and is fixed to CDD via disulfide bridge (C2179-C1633, highlighted yellow). (**G**) Putative kinesin-binding motif WD4 in α-herpesvirus large tegument protein (*24*) maps to HCMV pUL48 residues TQWPAM (purple), which reside on an exposed loop of pUL48’s CPD β barrel. (**H**) In each AU, an upper and lower pUL47/48 dimer stack to form a tetramer. TQWPAM is exposed on the upper pUL47/48 dimer. (**I**) Extensive interactions between pUL48-u and pUL48-l facilitate tetramer assembly. Left: pUL48-u shown as violet silhouette; interacting residues of pUL48-l shown as pink spheres. Right: pUL48-l shown as blue silhouette; interacting residues of pUL48-u shown as purple spheres. (**J**) Full atomic structure of VC1 PVAT and surrounding capsid. PVAT AU1 depicted in cylinder-stub. (**K** and **L**) pUL47-l drives inter-AU assembly through interactions with an adjacent AU’s pUL47-u (K) and pUL48-l (L). (**M** and **N**) SCP-VC1 PVAT interactions occur at each AU’s CBD helix bundle (M) and pUL48-l CPD (N). aa., amino acids.

Resolution of LTL was sufficient in our VC1 reconstruction to permit atomic modeling of pUL48 and pUL47 within LTL’s lower layer (LTL-lower) (Fig. 4, A to C). (We henceforth refer to LTL-lower copies of pUL47/48 as pUL47/48-l, and copies in LTL’s upper layer, or LTL-upper, as pUL47/48-u. In addition, while continuity between pUL48 CBDs and their respective head domains is visible in VC1, permitting definitive assignment of “u” and “l” copies, this is not the case in every configuration. We nonetheless maintain “u” and “l” nomenclature for pUL48 and pUL77 copies across all configurations, assigned to our best judgment, for the purposes of clarity and conciseness). Our atomic model of pUL48-l covers the C-terminal 798 residues (amino acids 1444 to 2241) of full-length pUL48’s 2241 residues (Fig. 4C). This region of the protein forms an elongated globular head with several structural elements/domains, which we assign as follows: coiled-coil motif (amino acids 1444 to 1556), containing a set of parallel helices pointing away from pUL48’s head; capsid-distal domain (CDD, amino acids 1557 to 1642, 1815 to 1956, and 1981 to 2034); capsid-proximal domain (CPD, amino acids 1643 to 1814, 1957 to 1980, and 2035 to 2079), containing a β barrel core; and linker–CBD (amino acids 2080 to 2241), composed of a 119-residue loop-rich linker and a pUL93-binding helix at pUL48’s C terminus that contributes to the CVSC helix bundle (Fig. 4D). For all copies of pUL48 visible in our VC1/NC1 reconstructions, an accompanying copy of pUL47 (of similar density strength to pUL48) can be seen closely bound, forming a pUL47/48 dimer. Our pUL47-l atomic model covers the N-terminal 521 residues (amino acids 5 to 521) of full-length pUL47’s 983 residues, to which we assign the following: apical domain (ApD, amino acids 1 to 82 and 483 to 520), at the apex of pUL47/48 dimer; spine domain (SpD, amino acids 83 to 221), forming the backbone of pUL47; linker-stabilizing domain (LSD, amino acids 222 to 324), adjacent to pUL48’s linker–CBD; and large-tegument–binding domain (LTBD, amino acids 325 to 482) (Fig. 4, B and D).

pUL47/48 dimerization is mediated through two interfacing regions, which combine for a buried solvent-accessible surface area of 1521.7 Å^2^. The major interface occurs between a helix-rich region of pUL47’s LTBD and pUL48’s coiled-coil motif, along with several residues of pUL48’s CDD (Fig. 4E). A second set of interactions occurs on the dimer’s opposing face, where pUL47’s LSD forms a pseudo-β-sheet interaction with a hairpin motif in the linker of pUL48’s linker–CBD (Fig. 4F). Curiously, the same hairpin motif harbors a cysteine residue (C2179) that forms an intramolecular disulfide bond with C1633 from a nearby CDD helix. Given the loopy, almost haphazard nature that characterizes pUL48’s linker—the 119-residue linker is coiled in a back-and-forth manner against the side of pUL48’s CPD and CDD—it is tempting to think that this hairpin motif, with a well-defined local structure facilitated by pUL47-binding and an intramolecular disulfide bridge, may be responsible for securing pUL48’s linker and tethering pUL48 close to the portal vertex.

### pUL47/48 dimerization is the basis of VC1 and NC1 large tegument layer architecture

Perhaps indicative of its stability, pUL47/48 dimer structure undergoes little change across different local environments, as evidenced in LTL-upper. Although LTL-upper density was of worse quality than LTL-lower, we could confidently rigid-body fit pUL47/48-l dimer and generate pUL47/48-u models with only minor local adjustments. In their entirety, our LTL models show that pUL47/48-l and pUL47/48-u from the same asymmetric unit stack to form a tetramer, notably in a manner that exposes the tryptophan motif TQWPAM on pUL47/48-u dimer (Fig. 4, G and H). Situated on an exposed loop of pUL48’s CPD β barrel, TQWPAM (amino acids 1809 to 1814) maps to the essential kinesin-binding motif WD4, conserved in α-herpesviruses’ large tegument protein and previously shown to be necessary for proper intracellular viral transport (Fig. 4G and fig. S7) (*24*). In contrast, TQWPAM on pUL47/48-l is buried within the pUL47/48-u and pUL47/48-l stacking interface, which is mediated exclusively by interactions between upper and lower pUL48 (Fig. 4I). We however found no homologous motif in HCMV pUL48 for the α-herpesvirus–conserved WD3 (fig. S7, A and E), a nonessential but kinesin-binding enhancing motif—this perhaps reflects that α-herpesviruses are distinctly neurotropic [therefore requiring greater processivity binding motorized cellular transport proteins (*20*, *57*)], whereas HCMV is not [HCMV is conditionally neurotropic (*58*, *59*)].

Interasymmetric unit interactions of VC1/NC1 LTL are heavily pUL47-mediated, with both upper and lower pUL47/48 dimers contributing to assembly (Fig. 4J). Specifically, pUL47-l’s ApD and SpD form bridging interactions with a left-adjacent asymmetric unit’s pUL47-u (Fig. 4K) and pUL48-l (Fig. 4L). Some interactions also appear between pUL47-l and its left-adjacent asymmetric unit’s pUL77-u, although these occur mainly between disordered loops and are not well-defined (fig. S6A). While the primary means of VC1/NC1 PVAT attachment to the capsid is through CBD helices that integrate with triplex-bound pUL93, additional interactions facilitated by small capsid proteins (SCPs), which sit atop major capsid protein (MCP) capsomers, likely confer stability to the top-heavy LTL. These SCP interactions occur between the CVSC helix bundle and P1 SCP (Fig. 4M), as well as between pUL48-l CPD and P6 SCP (Fig. 4N). Tangentially, the manner in which P1 SCP clamps down on the CVSC helix bundle may suggest that CVSC binding precedes SCP decoration during capsid maturation.

### Structures of partially tegumented portal vertices demonstrate PVAT plasticity

**Fig. 5.**
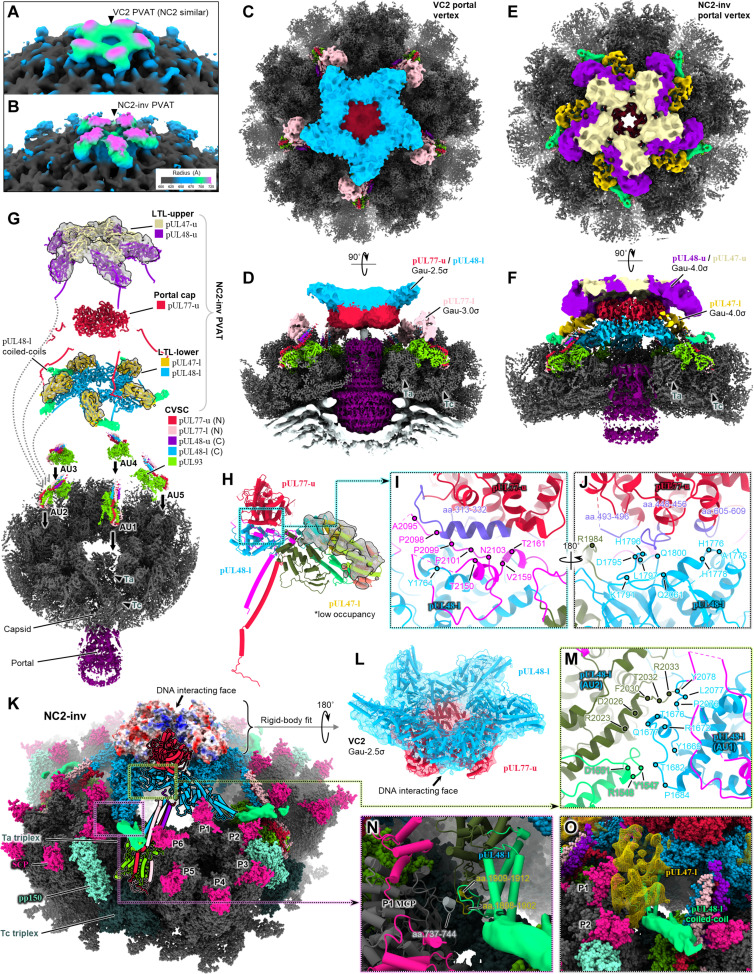
Structures of partially tegumented portal vertices demonstrate PVAT plasticity. (**A** and **B**) Global portal vertex-resolved reconstructions of VC2 virion (A) and NC2-inv NIEP (B) capsid. (**C** to **F**) Subparticle reconstructions of VC2 PVAT [(C) and (D)] and NC2-inv PVAT [(E) and (F)] segmented and colored as in (G). Contours chosen to best display overall structure features. (**G**) Exploded view showing components and layered organization of NC2-inv–decorated portal vertex. Low occupancy subunits displayed as models docked in mesh density. (**H**) NC2-inv pUL48-l interactions with pUL77 and low-occupancy pUL47-l (docked in mesh density), with pUL48-l and pUL47-l colored by domain. pUL47-l’s relative orientation to pUL48-l is unchanged from that observed in VC1/NC1 pUL47/48 dimers. (**I** and **J**) pUL48-l interactions with pUL77, with interfacing residue stretches in pUL77 colored purple, and interacting residues in pUL48-l denoted by colored dots. (**K**) Full atomic structure of NC2-inv pUL48/77 decamer and surrounding capsid (low-occupancy components of NC2-inv PVAT removed for clarity). PVAT AU1 depicted in cylinder-stub. pUL77 pentamer depicted as electrostatic surface, showing positively charged (blue), normally DNA-interacting residues facing outward, true to the inverted name. (**L**) NC2-inv pUL48/77 decamer, flipped 180° and rigid-body fit into VC2 PVAT density. (**M**) Inter-AU interactions in pUL48/77 decamer between adjacent pUL48-l copies. (**N**) pUL48-l (interacting residues yellow) contacts neighboring P1 MCP tower (interacting residues light gray). (**O**) pUL48-l and its low occupancy copy of pUL47-l (yellow mesh) fit into a valley-like cleft, likely increasing the stability of pUL48/77 decamer in NC2-inv. aa., amino acids.

Of the three remaining portal vertex reconstructions, we observed analogous PVAT density between a second pair of virion and NIEP PVAT configurations. We term these "virion configuration 2" (VC2) and "NIEP configuration 2" (NC2) (Fig. 5A, figs. S3H and S4, and movie S3). The remaining portal vertex reconstruction was generated from NIEP 3D classes containing a population of VC2/NC2-like PVATs that, much to our surprise, were inverted 180° vis-à-vis VC2/NC2 PVAT. We thus named this configuration "NIEP configuration 2, inverted" (NC2-inv) (Fig. 5B, fig. S4, and movie S4).

With the aid of our VC1 PVAT model, we interpreted the comparably lower resolution features of VC2 (henceforth also considered representative of NC2). Similar to VC1, VC2 has a pentameric portal cap composed of five copies of pUL77-u that harbor the genome terminus. However, unlike VC1 in which pUL77 does not interact with pUL48, we observe five lobes of density in VC2 resembling globular heads of pUL48 that bind portal cap’s outward face, forming a decameric pUL48/77 complex (Fig. 5, C and D). Other PVAT elements visible in VC1—including the remaining five heads of pUL48 and all 10 pUL48-bound copies of pUL47—are not visible in our VC2 reconstruction save for pUL77-l head domain, which in Gaussian-filtered maps appears docked (albeit with low occupancy) beside CVSC’s helix bundle (Fig. 5, C and D, and fig. S6E), in a position similar to that occupied by a putative pORF19 head domain in KSHV’s portal vertex (*43*). Despite the lack of ordered binding/low occupancy of a subset of pUL48 and pUL77 heads, VC2 CVSC still contains two copies of pUL48 CBD and two copies of pUL77 CBD, all well-resolved and of equivalent density strength. This indicates that VC2 portal vertices do retain a full complement of pUL77 and pUL48 and that although certain head elements may not be orderly bound, these are nonetheless flexibly linked in the vicinity of the portal vertex.

NC2-inv portal vertices similarly contain CVSCs with a full complement of pUL77 and pUL48 but otherwise exhibit different PVAT head occupancies and configuration (Fig. 5, E and F). Most apparently, NC2-inv contains a VC2-like pUL48/77 decamer that is flipped 180° relative to pUL48/77 decamer in VC2. Gaussian-filtered NC2-inv maps further reveal (i) very weak densities identifiable as pUL47 bound to pUL48 in the inverted decamer (Fig. 5, G and H) and (ii) the presence of a weak layer of density nested above the inverted decamer identifiable as a pentamer of pUL47/48 dimers (Fig. 5G). Despite these components’ low occupancy, both upper and lower copies of pUL47 bind pUL48 in a manner consistent with stable pUL47/48 dimer interactions described previously (Fig. 4, D to F). The second copy of pUL77 head domain is invisible in our NC2-inv reconstruction.

Intriguingly, the inverted pUL48/77 decamer in NC2-inv is much higher in resolution than in VC2. This enabled characterization of extensive interactions between pUL77 and pUL48-l, facilitated by pUL48-l’s linker and CPD (Fig. 5, H to J), and confirmed that the polarity of pUL77 pentamer is indeed inverted—namely, pUL77 pentamer’s positively charged DNA-interacting funnel faces away from the portal channel (Fig. 5K). Moreover, when inverted, NC2-inv’s pUL48/77 decamer model showed excellent fit within VC2’s PVAT density (Fig. 5L). We also identified interasymmetric unit pUL48-pUL48 interactions primarily between the CPD and CDD of adjacent pUL48-l subunits (Fig. 5M), which like the pUL77-pUL48 interface, are exclusive to pUL48/77 decamer and lack an equivalent in VC1/NC1 PVAT. However, whereas VC2 decamer lacks extensive contact with its underlying capsid (VC2 decamer is spatially localized only by portal cap binding of the genome terminus and flexible linker–CBDs anchored to the capsid), the inverted nature of NC2-inv’s decamer allows each pUL48-l copy to wedge within a valley-like topology between each asymmetric unit’s P hexon tower and adjacent CVSC helix bundle (Fig. 5, N and O). We speculate that this interlocking of pUL48-l with underlying capsid contributes to NC2-inv decamer stability and thus the improved resolution of features we observe. NC2 PVAT, lacking VC2’s portal cap interaction with genome terminus, underscores this logic, having an even higher degree of decamer smearing than VC2 (fig. S3H).

### pUL47/48 coiled-coil repeats manifest as filamentous PVAT densities

**Fig. 6.**
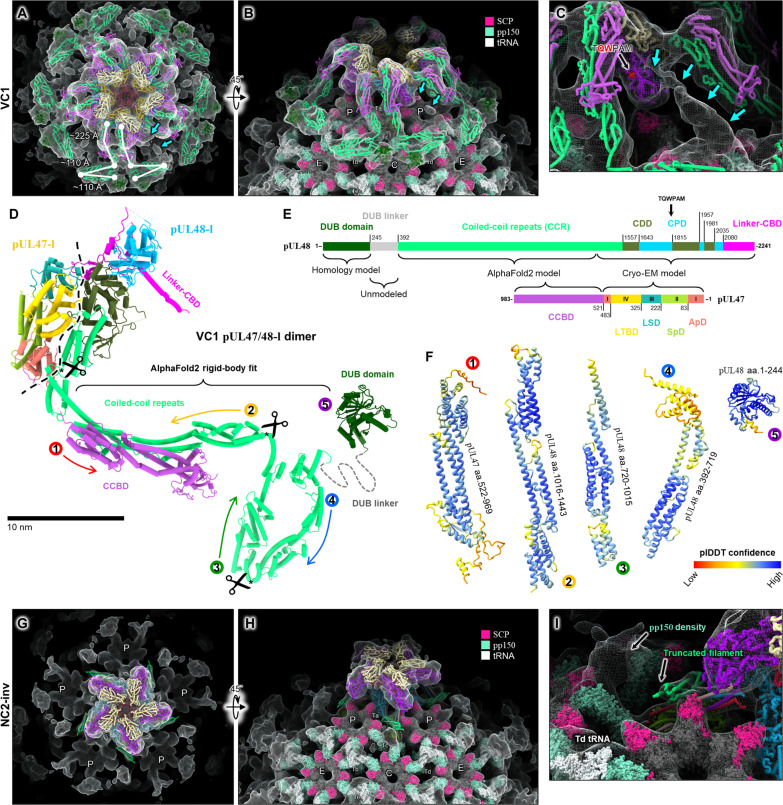
pUL47/pUL48 coiled-coil repeats manifest as filamentous PVAT densities. (**A** and **B**) Top (A) and side (B) views of VC1 PVAT with docked composite [i.e., AlphaFold2 (*61*) and cryo-EM] models. Ten sets of filaments arising from 10 pUL47/48 dimers decorate the portal vertex. Atomic models of surrounding SCP [PDB 5VKU (*38*)] and pp150 with bound tRNA [PDB 7LJ3 (*45*)] are shown to contextualize the filament environment. (**C**) Unassigned fibrillar density [cyan arrows; also in (A) and (B)] connects pUL48-u’s TQWPAM motif to its own CCR filament hairpin at low contours. (**D**) Composite pUL47/48 structure of cryo-EM atomic models and AlphaFold2-predicted models. (**E**) Schematic depicting domain organization and modeling of full-length pUL47 and pUL48. Colors correspond to (D). (**F**) AlphaFold2-predicted models of pUL47 C-terminal and pUL48 N-terminal fragments used for density-guided fitting. Models colored by predicted local-distance difference test (plDDT) confidence scores from low (red) to high (blue). (**G** and **H**) Top (G) and side (H) views of NC2-inv PVAT with docked composite models, as in (A) and (B). (**I**) NC2-inv pUL48-l harbors truncated CCR filamentous density. Unmodeled density adjacent to the truncated filament belongs to a pp150’s disordered C terminus. Mesh density in (A) to (C) and (G) to (I) is of VC1 and NC2-inv global capsid reconstructions, respectively. aa., amino acids.

We noticed tufts of filamentous density emanating from each pair of pUL47/48 dimers in VC1/NC1 PVAT (Fig. 2, D and F). These filaments were visible in their full length in our VC1/NC1 global capsid reconstructions, presenting as five pairs of 10 filaments splaying out from the portal vertex [Figs. 2 (A and B) and 6 (A to C)]. Filaments arising from pUL47/48-l and pUL47/48-u each contain initial parallel runs of long ~225-Å segments. These then kink in opposite directions, each forming a hairpin composed of two ~110-Å segments that lie draped atop the capsid, above portal-proximal SCP, triplex Tb, Tc, Td, C hexon, and their associated SCP (small capsid protein), pp150, and tRNA (*45*) elements (Fig. 6, A and B). Whereas average filament thickness varies by position from 20 to 35 Å in width, total length per filament is an enormous ~445 Å, sufficient to span one-third of a capsid or bridge the gap between capsid and viral envelope. These dimensions also resemble those of tuft-like features previously observed under transmission electron microscopy to extend from detergent-treated HSV-1 capsids, which were interpreted as arising from pUL48’s HSV-1 homolog, pUL36 (*60*).

To further investigate whether these filaments arise from unmodeled residues of pUL47 (amino acids 522 to 983) and pUL48 (amino acids 1 to 1443), we submitted full-length sequences of pUL47 and pUL48 to AlphaFold2 (*61*) for structure prediction (Fig. 6, D and E). AlphaFold2 results suggested elongated helix-rich structures for both unmodeled pUL47 C-terminal and pUL48 N-terminal fragments at high confidence intervals (Fig. 6F). In particular, pUL48 residues 392 to 1443 were predicted to form a long stalk-like structure characterized by multiple coiled-coil repeats, in agreement with previous in silico and biophysical analyses of the corresponding region in HSV-1 pUL36 (*37*). Using mostly rigid-body fitting, we fit both pUL47 and pUL48 predicted segments into VC1 filamentous density. Outward-extending filaments immediately proximal to the main PVAT structure contain a thick region, which we ascribed to pUL47 C terminus (coiled-coil binding domain, CCBD; amino acids 521 to 983) complexed with corresponding pUL48 coiled-coil repeat (CCR) region in antiparallel fashion (Fig. 6, D and E). To account for the filament hairpin, two kinks were introduced between adjacent pUL48 coiled-coil repeats at residues 720 and 1015 (Fig. 6D). We also observed a globular density near each filament’s distal end, into which we tentatively docked the predicted structure of pUL48’s N-terminal deubiquitinase (DUB) domain (*62*)—while tentative, this interpretation places pUL48 DUB in the general vicinity of triplex protein Tri2 (specifically Tri2 copies belonging to Tc triplex; Tri2 is HCMV pUL85), which was previously shown capable of pUL48 DUB binding (*63*). The resulting pair of filament models we constructed for each PVAT asymmetric unit are quasi-mirror images of each other (Fig. 6, A and B).

That the overall topology of pUL48 here, when traced from its C terminus in the CVSC to its filament-like N terminus, exhibits an “up” then “down” motif with respect to the capsid is consistent with models put forth in large tegument protein studies (*53*, *63*, *64*). However, we note that filament-capsid contact sites overall appear to be rather nonspecific and are less likely a result of well-defined capsid residues directing filament docking than the sheer length and bulk of filaments necessitating their resting on the capsid surface. (If binding were highly specific, one would expect to see well-resolved filamentous density at the hypothetical capsid binding sites). Such a loose “draping” of filaments on the capsid may be prognostic of their possible role as trafficking-related structures—kinesin and dynein, which pUL48 homologs are known to bind, frequently attach cargo via long filamentous structures thought to minimize steric hindrance between cargo and motor protein. Deletion of pUL48 amino acids 360 to 1200, which correspond to VC1/VC2 filaments, abrogates cytoplasmic distribution of otherwise infectious viruses (*63*).

Last, one feature of VC1 our models were unable to account for is a mysterious fibrillar density between pUL48-u’s TQWPAM motif and CCR filament hairpin (Fig. 6C). This fibrillar density is weak and only visible at high Gaussian filtering and low contour levels, suggesting a transiently bound, low-occupancy species. Considering TQWPAM’s possible role as a kinesin-interacting motif and in light of the recent evidence demonstrating assimilation of host cell kinesin in α-herpesviruses, we wonder whether this fibrillar feature could arise from captured copies of kinesin bound to pUL48 at low copy numbers.

### Truncated filamentous PVAT densities in NC2-inv portal vertices hint at polymorphic pUL48

VC2/NC2 portal vertices do not exhibit any features comparable to the notable filamentous densities seen in VC1/NC1. One plausible explanation for their absence could be that filaments in VC2/NC2 exist in an “extended” state [similar to that observed in detergent-treated HSV-1 capsids (*60*) mentioned previously], as opposed to a “retracted” or “capsid-draped” state as in VC1/NC1. Given VC2/NC2 PVAT density already exhibits flexible smearing due to minimal capsid interactions, it stands to reason that VC2/NC2 filaments would be more smeared due to even higher degrees of structural freedom. Alternatively, we cannot exclude that filaments may be altogether missing in VC2/NC2. Support for this lies in NC2-inv PVAT, where a short fragment of a filament can be seen extending from the apical coiled-coil motif of pUL48-l (Fig. 6, G and H). Fitting AlphaFold2’s prediction of pUL48 into this density suggests that CCR residues ~1267 to 1442 make up this fragment. Particularly, the cleanliness of the break in density beyond residue 1267 raises the possibility that pUL48-l CCR may be N-terminally truncated beyond residue 1267 (Fig. 6I). (Proteins that are merely flexible and/or disordered beyond a certain point will typically show some noise or low-resolution features near the juncture when filtered and/or displayed at low contours). Biochemical studies have hinted at the existence of variably truncated large tegument protein species in herpesviruses (*65*, *66*). These observations conjecture polymorphic pUL48 as an additional layer of heterogeneity to PVAT configuration.

### pUL47/48 dimers account for portal-biased tegumentation of penton vertices

**Fig. 7.**
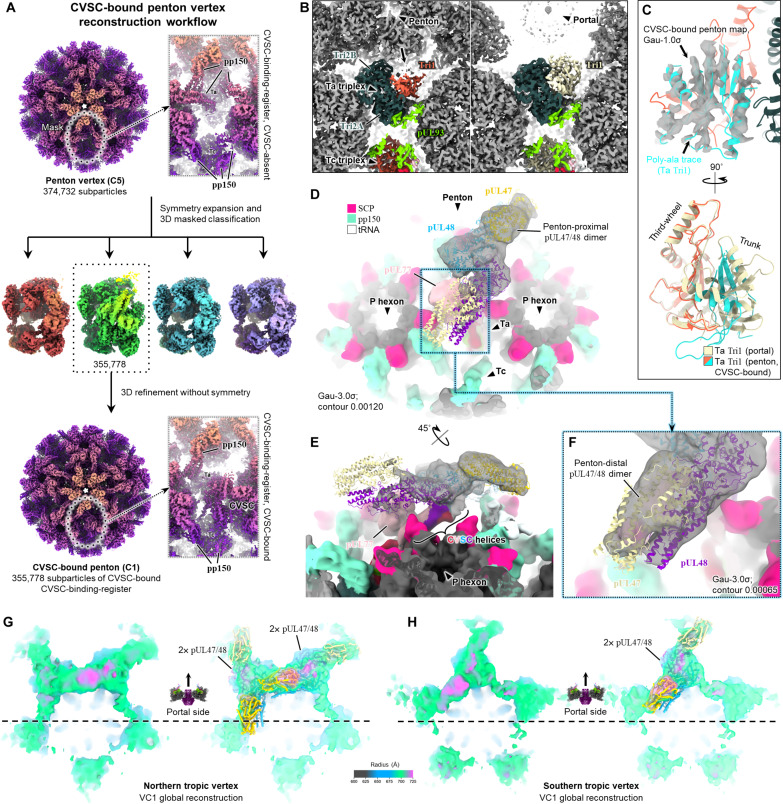
Structure of the penton vertex CVSC-binding-register. (**A**) Workflow for generating the C1 reconstruction of a penton vertex with one CVSC-bound CVSC-binding-register. (**B**) Comparison of Ta triplex underneath penton vertex CVSC (left) and portal vertex CVSC (right) reveals differences in density in penton-proximal Tri1’s trunk domain (black arrow). Both Ta triplexes are rotated ~120° counterclockwise relative to non–CVSC-bound Ta triplexes. (**C**) Filtering the CVSC-bound penton vertex map permits a poly-alanine backbone trace of penton-proximal CVSC-bound Ta Tri1’s trunk domain (~amino acids 40 to 160), which when superposed with portal-proximal Ta Tri1, highlights conformational rearrangement of penton-proximal CVSC-bound Ta Tri1’s trunk domain. (**D** and **E**) Globular head-like tegument densities extending from CVSC helices are visible above the CVSC-binding-register in filtered CVSC-bound penton vertex maps at low contours. These fit two docked copies of pUL47/48 dimer. (**F**) The more penton-distal pUL47/48 dimer has noticeably weaker density. (**G** and **H**) Docking models of penton CVSC with its associated pUL47/48 dimers into the northern (G) and southern (H) tropic penton vertices show that pUL48’s globular head and pUL47 account for the preferred tegumentation of portal-proximal CVSC-binding-registers at penton vertices. Dashed lines represent equatorials delineating portal-proximal and portal-distal sides of each penton vertex.

With insights from our structural analyses of PVAT in hand, we sought to investigate the structural basis of portal-biased tegumentation at penton vertices. To do so, we classified the five CVSC-binding-registers surrounding each penton vertex as in our previous KSHV work (*43*) and reconstructed a CVSC-bound penton vertex with one CVSC-bound CVSC-binding-register at 3.02-Å resolution [Fig. 7A, figs. S3 (F and G) and S4, and table S1]. Similar to the portal-proximal Ta triplex, penton-proximal CVSC-bound Ta triplex here exhibits the same 120° counterclockwise rotation relative to non–CVSC-bound Ta triplexes that has been previously documented (Fig. 7B) (*42*–*44*, *46*, *67*). However, we noted an additional conformation change in the first ~160 residues of triplex protein Tri1 (HCMV pUL46) in penton-proximal CVSC-bound Ta triplex that has not been seen in Tri1 of portal-proximal Ta triplex nor any other Tri1 position or homolog to our knowledge (Fig. 7, B and C). We also identified one copy of pUL77 (fig. S6F) as well as two oblong, dual-lobed densities that are visible in filtered maps above CVSC (Fig. 7D). Each dual-lobed density satisfactorily fits a model of PVAT pUL47/48 dimer without any morphing of the dimer, although the penton-distal dimer has visibly weaker density (Fig. 7, E and F).

Rigid-body docking both pUL47/48 dimers with penton CVSC en bloc into a global portal-resolved capsid reconstruction shows that portal-proximal tegument densities above northern tropic penton vertices fit two adjacent CVSC complements (Fig. 7G) whereas those of southern tropic penton vertices easily fit one CVSC complement (Fig. 7H). This confirms that portal-biased tegumentation of penton vertices arises from CVSC binding and demonstrates that pUL48’s core fold and association with pUL47 are conserved even within the penton vertex’s local environment (fig. S8). We point out here that our global portal-resolved capsid reconstructions are the C5 symmetrized averages of many individual virus particles and as such are faithful representations of per vertex CVSC binding tendencies on a population-wide level. They do not, however, recapitulate the variable nature of CVSC occupancy in a per binding-register sense, which in reality yields several possible permutations of CVSC binding at penton vertices, as has been recently explored in-depth by our group and others in HCMV and KSHV (*43*, *46*). Nonetheless, prior granular analyses consistently show that the most common penton vertices are permutations with two adjacent CVSCs bound and one CVSC bound, mirroring the tegumentation we observe at portal-proximal CVSC-binding-registers in northern tropic and southern tropic penton vertices, respectively. These prior analyses further explain the weak tegument densities on portal-distal CVSC-binding-registers, which are likely the smeared densities of low occupancy pUL47/48 dimers arising from low frequency permutations of CVSC-bound penton vertex. [This is quite a complex subject, so the takeaway is that portal-biased CVSC binding consistent with portal-biased tegumentation documented here has been observed in β- and γ-herpesviruses. Whether this is true for α-herpesviruses remains to be rigorously examined, although α-herpesviruses appear to exhibit much higher base levels of CVSC occupancy at all capsid vertex registers (*12*, *39*, *41*).] Last, neither tegument configuration in northern or southern tropic penton vertices resemble VC1 or VC2 or any of their NIEP portal vertex derivatives. Despite the large number of subparticles used in our CVSC-bound penton vertex reconstruction, tegument density here is still relatively low resolution, likely due to the flexibility of CVSC head domains, which, in the absence of sufficient occupancy at all five CVSC-binding-registers, are unable to form stabilized ring structures as above the portal vertex.

## DISCUSSION

Our machine learning–based approach to processing cryotomograms permitted direct observation of truly single-particle data of HCMV virions and NIEPs, revealing a portal-biased scheme of tegument acquisition. Further cryo-EM analyses corroborated this observation and revealed distinct configurations of PVAT reflecting varying degrees of ordered tegument assembly. Modeling these assemblies revealed the mechanistic bases of genome containment by pUL77, networks of interactions among PVAT components, and characterization of 45-nm-long pUL47/48 filaments. Our results offer a structural perspective for evaluating the substantial body of biochemical and genetic work on the herpesvirus tegument and in particular the capsid-associated tegument proteins conserved across all three subfamilies of herpesviruses.

A principal focus of tegument work is with respect to herpesvirus assembly and the physical organization of tegument components. As the largest tegument protein, the aptly named large tegument protein (e.g., HCMV pUL48, HSV-1 pUL36, and KSHV pORF64) has attracted much attention as a probable link between the rigid symmetry of the capsid and the pleomorphic tegument, on account of its C-terminal anchoring to the capsid surface and multipartite domains’ ability to bind, and thus nucleate, a number of tegument proteins (*10*, *11*, *15*, *64*, *68*). Inner tegument protein (e.g., HCMV pUL47, HSV-1 pUL37, and KSHV pORF63) is an important large tegument protein binding partner and effector (*62*, *69*) with similarly diverse interactions, ranging from the disordered C terminus of β-herpesvirus–specific pp150 (*70*) to α-herpesvirus–specific envelope glycoproteins (*26*). Our atomic description of the pUL47/48 dimer presents structural confirmation of pUL47’s suspected 1:1 association with pUL48 and affirms the analogous association of their herpesviral homologs. However, prevailing evidence relegating the binding interface of pUL47/48 dimer to the N-terminal one-third of pUL48 and C-terminal one-third of pUL47—a notion also held with their homologs (*62*, *69*)—do not appear consistent with what we observe in our structures, where the bulk of interactions occur between pUL48’s C-terminal globular head and pUL47’s N-terminal fragment [Figs. 4 (D to F) and 6E]. That this pUL47/48 dimer arrangement is the only arrangement we observe and appears conserved in all configurations and local environments where pUL47 is present (i.e., VC1, upper and lower layers; VC2, upper and lower layers; NC2-inv, upper and lower layers; CVSC-bound penton registers, both pUL47/48 dimers) is perhaps indicative of both its specificity and stability. We emphasize this is not to say that prior characterizations of the pUL47/48 dimer interface are necessarily incorrect, given VC1/VC2 are the only configurations we obtained in which the supposed N- and C-terminal interacting regions of pUL48 and pUL47, respectively, are even visible (i.e., the filamentous densities) and limited to intermediate resolution at that. To be clear, our density-guided fitting of AlphaFold2 segments here also does not support the previous evidence, but given that (i) these regions are not visible in our reconstructions of other configurations containing pUL47/48 dimer and (ii) there may be other PVAT configurations not represented here, we cannot exclude that alternate interactions may exist between pUL47 and pUL48 apart from what we observe.

Underscoring the theme of variable tegument organization, our discovery of multiple PVAT configurations in enveloped particles highlights the vast conformational space that PVAT structures occupy (fig. S8). While our data do not speak to the function of observed configurations, their existence is informative in several ways. One, that virion and NIEP PVAT configurations are generally mirrored in distribution, organization, and composition (this also includes their global capsid tegumentation; NC2-inv is a special case we address later) suggests that acquisition of capsid-associated tegument proteins and the emergence of PVAT variation does not discriminate between capsids that are genome-containing or not, at least for particles that successfully egress host nuclei. Two, the permuting sets of interactions we observed among PVAT components in different configurations define a basis of molecular modularity that explains PVAT’s organizational plasticity. Three, we note that among all resolved configurations, all CVSC components of the portal vertex are present, yet their globular head domains and binding partners display variable occupancy in certain configurations (e.g., VC2 and NC2-inv). This reflects a degree of reversibility in the binding of PVAT componentry, which raises the possibility of an equilibrium of sorts among PVAT configurations. Pertinently, single-particle classification and reconstruction inherently selects for groups of particles that are similar and stable to achieve (hopefully) high resolution. Although we discarded less than 50% of extracted PVAT subparticles, our reconstructions are therefore best understood as corresponding to minima in PVAT’s configurational landscape, which, for reasons unknown, is slightly biased in both virion and NIEPs toward the more labile “configuration 2”–type structures. There well could be virus particles of intermediate configurations in partial states of PVAT (dis)assembly not captured in our structures. That said, these are likely few, as most subparticles that did not contribute to final PVAT reconstructions still resembled a resolved configuration (fig. S2).

**Fig. 8.**
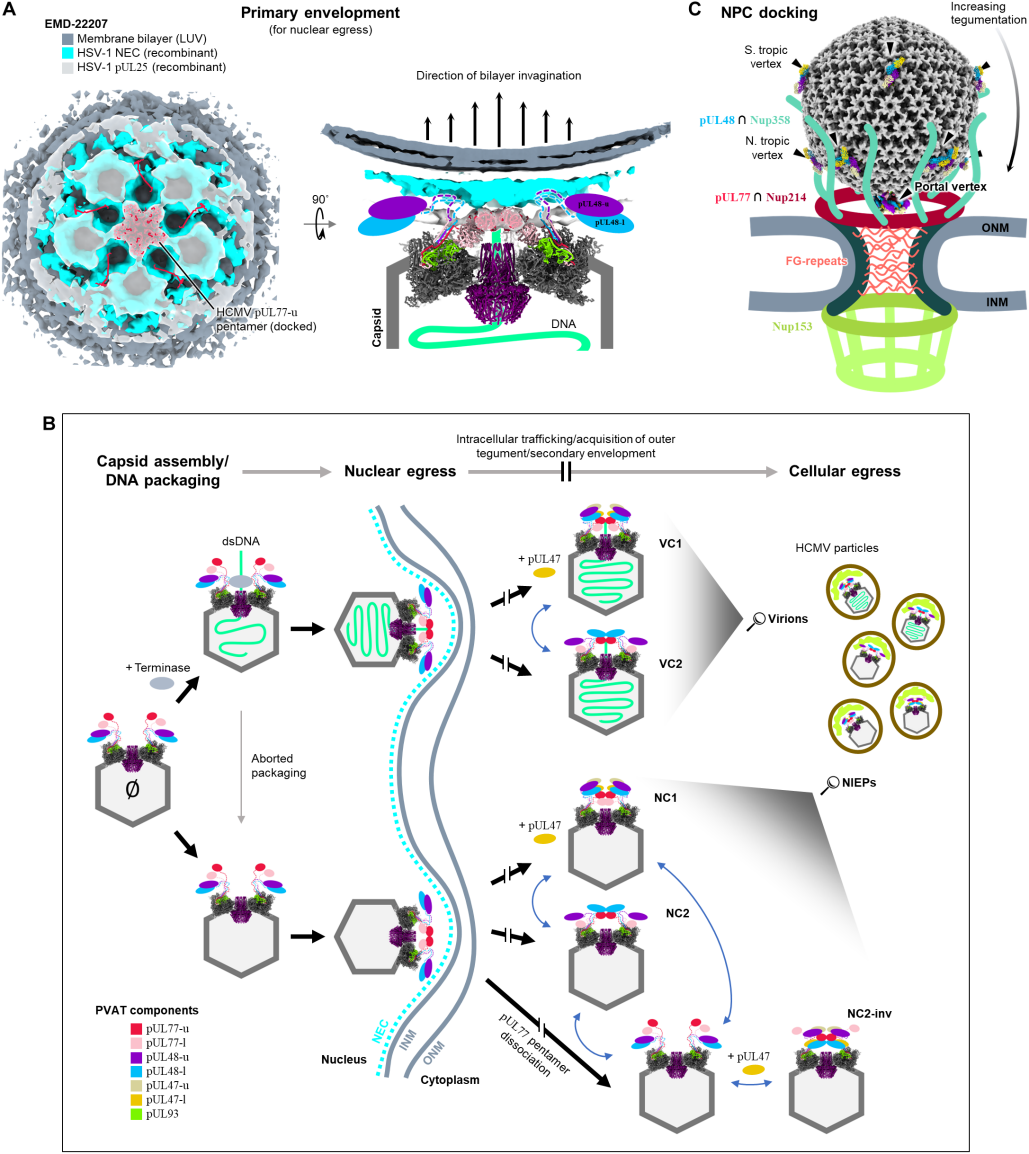
A highly configurable tegument may facilitate capsid traversal through cellular architecture. (**A**) Density map is a subtomogram reconstruction (EMD-22207) from an in vitro large unilamellar vesicle (LUV) system previously used to study herpesvirus primary envelopment during nuclear egress. Recombinantly expressed HSV-1 pUL25 was found to form star-shaped clusters of density when introduced to an HSV-1 NEC lattice seeded on the LUV surface (*16*). Capsid-bound pUL25 was proposed to initiate NEC-mediated budding. Our capsid-derived HCMV pUL77 pentamer model shows good fit with star-shaped HSV-1 pUL25 density. Rotated side view shows a cartoon illustrating how linker–CBDs might allow pUL48 heads to flexibly fold aside, thus permitting pUL77 pentamer-NEC interactions required for primary envelopment. (**B**) Flowchart illustrating the proposed evolution of PVAT structure during virus maturation. Our data show enveloped virus particles contain a heterogeneous population of PVAT states, which analyses suggest may be an equilibrium of reconfiguring states, as indicated by blue double-headed arrows. Break bars in arrows represent processes not illustrated and for which PVAT structure is unknown and cannot be inferred. Nuclear capsids as illustrated here reflect the current understanding of nascent virion and NIEP formation, including the attachment of CVSC components to nuclear capsids, and NEC-mediated primary envelopment. (**C**) Diagram illustrating how portal-biased tegumentation may facilitate proper portal vertex orientation during NPC docking via tegument-nucleoporin interactions.

A parallel focus of tegument studies revolves around herpesvirus-host interactions. From viral genome to packaging delivery, herpesviruses navigate a series of transitions largely dictated by eukaryotic cell architecture. These include (i) for virion maturation and release: primary envelopment (nuclear egress), outbound microtubule (MT)–dependent transport, budding through the Golgi apparatus, and secondary envelopment (cellular egress); and (ii) upon infection: fusion with the host membrane/loss of outer tegument, minus-end directed MT transport to the centrosome, plus-end directed MT transport to the nucleus, NPC docking, and finally, pressurized ejection of genome through the NPC. Various protein components in PVAT come to the forefront in each of these processes (*9*, *15*, *16*, *19*–*24*, *27*, *28*, *31*). Therefore, central to proper viral function is how to permit necessary PVAT reconfiguration in an efficient manner without PVAT detachment from or gross disassembly of the portal vertex, lest packaged genome be prematurely released. Although this current study visualized only extracellular virus particles, our characterization of PVAT architecture and its plasticity inform a plausible solution to this dilemma by means of layered PVAT protein interactions and a prevalent head–linker–CBD protein motif. We briefly consider these strategies within the context of our structures and recent literature in two snapshots of the herpesvirus life cycle below (Fig. 8).

First, nuclear egress represents the initial encounter of higher-order cell structure for nascent herpesvirus capsids. Too large to transit NPCs, capsids bound for cytoplasm must first bud through the inner nuclear membrane (INM) before deenvelopment across the outer nuclear membrane (ONM) (*71*). Best studied in HSV-1, budding at the INM or primary envelopment is facilitated by an ordered coating of nuclear egress complex (NEC) composed of herpesvirus-conserved HSV-1 pUL31 and pUL34 (*72*). NEC binding by capsid-bound pUL25 (HSV-1’s pUL77 homolog) is thought to initiate spontaneous INM deformation and scission (*73*), a notion supported by in vitro cryo-ET reconstructions of synthetic-vesicle–bound NEC complexed with recombinant pUL25 (*16*). Our in situ model of pUL77 pentamer fits remarkably well into pUL25 density from said reconstruction (Fig. 8A), strongly indicating that pUL25 pentamerization observed in complex with NEC indeed reflects pUL25’s actual capsid-bound configuration. This has several interesting stipulations with respect to PVAT configuration at this stage. One, the outward (i.e., non–DNA-retaining) face of pUL77 pentamer is almost certainly the NEC-interacting face, lest captive genome be released. Primary envelopment must then occur only given presentation of the nominal “genome captive” orientation of pUL77 pentamer (Fig. 8A, right). Whether genome is in fact captive is irrelevant, as NIEPs evidently egress nuclei successfully. [As a side note, that the inverted pUL77 pentamer of NC2-inv PVAT exists suggests nominal pentamer orientation need not be stringently maintained in NIEPs postnuclear egress. Because the entire pUL77 pentamer and five attached copies of pUL48 (i.e., the pUL48/77 decamer) cannot as an ensemble flip 180° without dissociating and then reconstituting (as each of these subunits are anchored by their respective linker–CBDs, and the linkers are insufficiently long to permit an ensemble flip), this further suggests that PVAT configurations may exist in some sort of equilibrium and be interconvertible (Fig. 8B).] Two, steric interference by non-pUL77 PVAT elements must be minimized at the pUL77-NEC interface. Given CVSC constituents including large tegument protein are already present in nuclear capsids (*74*–*76*) (also unpublished data), pUL48’s head–linker–CBD organization conceivably permits declashing of its not insubstantial head away from pUL77 pentamer, thereby facilitating proper presentation of the pentamer’s NEC-binding interface (Fig. 8, A and B).

Second, upon gaining entry to a new host cell, herpesvirus capsids must once again overcome the nuclear envelope to deliver genetic payload to the host nucleus. Truncation studies have shown that a herpesvirus-conserved nuclear localization signal near the DUB domain of large tegument proteins route incoming capsids to NPCs (*63*, *77*, *78*), where docking occurs in preparation for genome ejection through the NPC. Substantial evidence suggests HSV-1 pUL25 interacts with nucleoporin Nup214, which forms NPC’s cytoplasmic ring, whereas HSV-1 pUL36 interacts with Nup358, which gives rise to NPC’s MT-interacting cytoplasmic filaments (*31*, *79*–*81*). Correct orientation of portal vertex to NPC is critically important, as aberrantly oriented capsids do not further progress toward genome ejection (*28*). Again, our structures offer a framework for these data. First, the portal-biased tegumentation of capsid vertices by CVSC, of which pUL77 and pUL48 are major components [Figs. 2 (A to C) and 7 (H and I)] lends well to proper orienting of portal vertex to NPC by CVSC-Nup214/358 interactions (Fig. 8C). That Nup358 is MT-interacting and pUL48 binds MT-transport proteins elaborates this scheme—a seamless transfer of capsid from cytoskeletal MT elements to NPC, mediated by pUL48, can be envisaged. Furthermore, nominal genome ejection of NPC-docked HSV-1 capsids is contingent upon exposure and subsequent triggering of three C-terminal pUL25 residues (*15*), the equivalent of which encircle the outward-facing (i.e., NPC-facing) pore of pUL77 pentamer (Fig. 3K, dashed circles) in tantalizing and, pending declashing of any obstructing pUL48 globular heads, quite accessible fashion. We surmise that the configurational flexibility offered by PVAT structure would once again facilitate these tegument-choreographed transitions directing NPC docking to genome delivery.

In summary, we present here the elusive structures of the herpesvirus tegument and provide an initial framework for future structure-function analyses of tegument components. Our results inform herpesvirus assembly and host interactions and raise several follow-on questions for each. Most interesting to us is how do herpesvirus portals effect long-range portal-biased acquisition of tegument at capsid sites tens of nanometers away and whether and how the PVAT states we resolve play roles during viral infection. Intracellular investigations are key to parsing these mysteries, which we expect the imminent maturation of cryogenic-focused-ion-beam milling and tomography to greatly benefit. Technically, our employment of artificial intelligence (AI)–enhanced cryo-ET, high-resolution cryo-EM, and accurate structure prediction showcases the power of integrated analyses leveraging multiple modalities of structure investigation across multiple resolutions, which in our view is the thrilling future of structural biology.

## MATERIALS AND METHODS

### Viruses and cell lines

We drew on two previously prepared batches of isolated HCMV virions for this investigation. We have previously described these protocols in detail (*38*, *45*), but we briefly summarize them here. In the first batch (henceforth "batch-1"), human fibroblast MRC-5 cells [American Type Culture Collection (ATCC) CCL-171] were grown in Dulbecco’s modified Eagle medium (ATCC 30-2002) supplemented with 10% fetal bovine serum (FBS) and then infected with HCMV strain AD169 (ATCC VR-538). Culture medium was collected 8 days post-infection (DPI) and centrifuged at 10,000*g* for 12 min to remove cell debris. We collected supernatant and centrifuged at 80,000*g* for 1 hour to pellet HCMV particles. The pellet was then resuspended in phosphate-buffered saline (10 mM PBS, pH 7.4), loaded on 15 to 50% (w/v) sucrose gradient, and centrifuged at 100,000*g* for 1 hour. Virus particle–containing light-scattering bands were collected, diluted with PBS, and then pelleted at 80,000*g* for another hour. For the second batch (henceforth "batch-2"), MRC-5 cells were grown in Eagle’s minimum essential medium (ATCC 30-2003) supplemented with 10% FBS and then also infected with HCMV strain AD169. Culture medium was collected 8 DPI and then similarly centrifuged to obtain a virus particle–containing pellet. Final pellets from both batches were resuspended in PBS for imaging-specific preparation (described below).

### Cryo-ET imaging and tomographic reconstruction

We previously described in detail the cryo-ET of HCMV particles (*50*). Briefly, HCMV particles from batch-1 were mixed with 5-nm gold beads and applied onto freshly glow-discharged Quantifoil Holey Carbon Grids (2/1) before plunge freezing into liquid ethane. Tilt series were acquired using the SerialEM software suite (*82*) on a Thermo Fisher Scientific Titan Krios electron microscope operated at 300 kV and equipped with a Gatan imaging filter, Gatan K2 Summit direct electron detector, and Volta phase plate (VPP). Before collecting each tilt series, we advanced, then preconditioned the VPP by electron illumination at a dose of 12 nC for 60 s to ensure a phase shift of ~0.3π. Movies were recorded with the K2 Summit camera operated in counting mode at 5 frames/s with a dose rate of ~8 to 10 *e*^−^ per pixel/s. Tilt series were collected from −66° to +60° at 2° intervals at 53,000× nominal magnification (for a calibrated pixel size of 2.6 Å) with a cumulative electron dose of approximately 100 e^−^/Å^2^. Defocus was targeted to −0.6 μm. The collected dataset we used to treat with our missing-wedge recovery algorithm (described below) consisted of 11 tomograms.

Collected movies of raw tilt series were aligned and averaged using MotionCor2 (*83*) to generate a single micrograph for each tilt angle. Tilt series were aligned by tracking gold fiducial beads, and tomograms were reconstructed using the IMOD 4.11 software package (*84*) implementing the simultaneous iterative reconstruction technique method.

### Machine learning–based enhancement of tomograms by IsoNet

Full usage details of IsoNet and a complete description of its workflow have previously been elaborated (*49*). Here, we train IsoNet specifically on our HCMV cryo-ET dataset. Briefly, we first generated tomogram STAR files for all 11 tomograms using the IsoNet function isonet.py prepare_star. We then proceeded to downscale reconstructed tomograms using isonet.py rescale, resulting in 6×-binned tomograms with a pixel size of 15.6 Å per pixel. Because tomograms were acquired using a VPP, we did not perform the usual step of CTF deconvolution. A tomogram-wide mask was created using isonet.py make_mask, which automatically targets and identifies feature-rich regions of the tomogram for subsequent subtomogram extraction and neural network analysis.

Four hundred subtomograms (96 × 96 × 96 pixels) centered on feature-containing masked regions were then randomly extracted from the 11 tomograms using isonet.py extract. Using isonet.py refine, we fed these subtomograms as raw inputs to generate the initial neural network for dataset-optimized missing-wedge correction and denoising. By default, 30 iterations of neural network and data refinement occur, with each successive iteration implementing both the previous iteration’s network and data output as new inputs. Denoising—in short, artificially introducing noise into our dataset to promote neural network discernment of noise within signal data—was gradually increased from none in the first 10 iterations to 0.2 in the final five iterations. The final trained neural network, specifically optimized on our entire set of tomograms, was then applied to binned individual whole tomograms using isonet.py predict to obtain final denoised and missing-wedge–corrected tomograms.

### Cryo-EM imaging of HCMV particles

To acquire a dataset sufficient to resolve the large and complicated structures of the portal-associated tegument, we exhaustively imaged both HCMV batch preparations over multiple imaging sessions. Our general workflow is as follows. 2-μl aliquots of resuspended virus pellet were pipetted onto Quantifoil Holey Carbon Grids (2/1). Plunge freezing of both batches was carried out in liquid ethane. Vitrification of batch-1 was performed immediately after sample treatment with 1% NP-40 under optimized conditions in an FEI Vitrobot Mark IV operated at 100% humidity with a blot time of 20 s. Batch-2 was vitrified using a manual plunger with a blot time of 6 s. Cryo-EM imaging was performed using a Thermo Fisher Scientific Titan Krios electron microscope operated at 300 kV, equipped with a Gatan imaging filter and Gatan K2 Summit direct electron detector. Earlier datasets from batch-1 were collected using the Leginon software suite (*85*) with K2 Summit camera operated in electron counting mode and magnification set to a calibrated 31,120×, giving a pixel size of 1.61 Å per pixel. We used an electron dose rate of ~7 *e*^−^ per pixel/s on camera, which corresponds to a dose rate of ~2.7 *e*^−^/Å^2^/s on specimen. A total of ~12,000 movies were collected with movies recorded at 4 frames/s and 14 s per movie. Later datasets from batch-2 were recorded on the K2 Summit camera operated in super-resolution mode running SerialEM (*82*) and 105,000× nominal magnification, yielding a pixel size of 1.36 Å per physical pixel (0.68 Å per pixel in super-resolution). Electron dose rate was set to ~11 *e*^−^ per pixel/s on camera, corresponding to an on-specimen dose rate of ~5.9 *e*^−^/Å^2^/s. Movies were collected at 5 frames/s for 8 s per movie, and a total of 83,488 movies were collected. Following cryo-EM imaging, movie stacks were drift-corrected and averaged using MotionCor2 (*83*) to generate corresponding micrographs. Micrograph defocus values were determined using CTFFIND3 (*86*).

### Particle picking, separation of virions and NIEPs, and initial icosahedral reconstruction

We observed both HCMV virions and NIEPs in our micrographs. In our combined batch-1 dataset, we manually boxed out 19,309 virions and 34,191 NIEPs using 3dmod in IMOD (*84*), using a box size of 1024 × 1024 pixels. In our combined batch-2 dataset, we performed semiautomated particle picking without first distinguishing between virions and NIEPs. We began by first picking 43,666 particles (box size of 1280 × 1280 pixels) by hand from 16,522 micrographs again using 3dmod. Coordinates of these 43,666 particles were then input to Topaz (*87*) as training data to facilitate neural network–accelerated particle picking. Topaz automated picking was then deployed on all 83,488 batch-2 micrographs (each 16×-binned to further accelerate particle identification) to generate target particle coordinates, which were then piped into RELION 3.1 (*88*) for particle extraction. A total of 313,507 particles were extracted from the combined batch-2 dataset.

Whereas virions and NIEPs were manually curated and separated in the combined batch-1 dataset, we addressed virion and NIEP separation in the much larger combined batch-2 dataset through a 3D classification workflow. Briefly, we partitioned the batch-2 dataset into several subsets and used RELION to generate initial icosahedral reconstructions with I3 symmetry from 4×-binned particles for each subset. We then performed 3D classification without orientation search, which yielded classes in which virions and NIEPs could easily (and quickly) be distinguished. While not necessary for virion and NIEP curation, we also generated a consensus I3 icosahedral reconstruction using 2×-binned particles from our combined batch-1 dataset, which we use as a reference for subsequent subparticle extraction (fig. S2).

### Symmetry relaxation and capsid vertex subparticle extraction

To extract fivefold capsid vertex subparticles for both virions and NIEPs, we used a subparticle extraction and reconstruction protocol we optimized for large viruses, which we established in a previous paper (fig. S2) (*42*). Briefly, we expanded initial icosahedral reconstructions (with I3 symmetry) using RELION’s relion_particle_symmetry_expand script. This generated a symmetry expanded data STAR file in which all 60 symmetry-related orientations are fully described for each virus particle. Each orientation has three identifying Euler angles represented as parameters within the STAR file: rot (_rlnAngleRot), tilt (_rlnAngleTilt), and psi (_rlnAnglePsi). We noted that there are five redundant orientations relative to each vertex that differ only in the orientations’ rot angles (rotation about the *z* axis). Given this, we assigned 60 orientations into 12 groups with 5 orientations per group such that orientations within a group have different rot angles but identical tilt and psi angles. Last, we randomly selected one orientation per group as the de facto orientation of the vertex, thus generating one orientation for each vertex, yielding a total of 12 unique orientations (vertices) out of 60 icosahedrally related orientations. Using these orientations, the 2D positions of all 12 vertex subparticles of the icosahedral viral capsid were calculated on each viral particle image, based on previously defined equations (*42*). We then extracted these vertex subparticles using relion_preprocess with a box size of 300 × 300 pixels for batch-1 and 360 × 360 pixels for batch-2.

### Identification of portal vertex subparticles through 3D classification

We performed RELION 3D refinement on previously extracted capsid vertices with C5 symmetry using only local search for orientation determination. References were generated using relion_reconstruct and filtered to 40-Å resolution for refinement. To overcome the well-documented depth-of-focus problem for enormous virus particles such as herpesviruses, we performed per-particle defocus refinement and beam tilt refinement using relion_ctf_refine. Because our particles originated from two different datasets (batch-1 and batch-2), we additionally performed magnification correction also using relion_ctf_refine. We then subjected the resulting particles to a second iteration of refinement followed by defocus, beam tilt, and magnification refinement.

To separate portal vertex subparticles from penton vertex subparticles, we performed 3D classification with fivefold symmetry while limiting the resolution to 8 Å. No orientation and translation search was performed (using RELION argument skip_align). Resulting 3D classes exhibiting a unique class different from other classes were readily identifiable as the capsid’s unique portal vertex (fig. S2). To maximally include possible portal vertex subparticles in the portal vertex class, we combined all particles appearing in the portal vertex class from iterations 15 to 50 (out of 50 total iterations per classification). These efforts netted 58,930 NIEP portal vertex subparticles and 244,813 virion portal vertex subparticles.

### 3D classification and reconstruction of virion PVAT

We noted proteinaceous tegument densities atop both virion and NIEP portal vertex subparticles. To analyze structures of virion PVAT, we imposed a spherical mask on our 3D class of virion portal vertex subparticles encompassing proteinaceous densities of PVAT and a bordering region of underlying capsid (fig. S2). This enabled us to perform focused 3D classification targeting the proteinaceous capping densities, which we carried out without orientation search and with a tau factor (regularization parameter) of 8. Resulting 3D classes revealed two ordered subtypes or configurations of virion PVAT, which we termed VC1 and VC2. VC1 classes consisted of a large complement of PVAT proteins with distinguishable secondary structure features. Generating the best resolution reconstruction of VC1 required keeping VC1 portal vertex subparticles of good quality while discarding portal vertex subparticles with flexible or disordered PVAT. To do so, we performed successive rounds 3D classification without orientation search, yielding a final curated population of 35,055 VC1 portal vertex subparticles. We performed 3D refinement with local orientation search on this curated population, resulting in a final VC1 portal vertex subparticle reconstruction with C5 symmetry, estimated at 3.50-Å resolution at the 0.143 gold-standard Fourier shell correlation (FSC) criterion [figs. S3 (A and G) and S4 and table S1].

VC2 classes had a noticeably smaller complement of PVAT proteins with a greater degree of variability in density. To generate the best reconstruction of VC2, we chose the VC2 class demonstrating strongest PVAT density to perform 3D refinement with local orientation search. This resulted in a final VC2 portal vertex subparticle reconstruction with C5 symmetry estimated at 3.27-Å resolution at the 0.143 FSC criterion [figs. S3 (B and G) and S4 and table S1]. We do note that because these subparticle reconstructions include PVAT’s underlying capsid structure, our estimated resolutions are reflective of the entire reconstruction and not just the PVAT, which is of varying degrees of lower resolution in our reconstructions.

### 3D classification and reconstruction of NIEP PVAT

To analyze structures of NIEP PVAT, we imposed a spherical mask on our 3D class of NIEP portal vertex subparticles encompassing a region analogous to that of our mask in virion portal vertex subparticle processing (fig. S2). We then performed focused classification targeting this region without orientation search and a tau factor of 8. Resulting 3D classes revealed three distinct populations of NIEP PVAT, which we termed NC1, NC2, and NC2-inv (corresponding to virion PVAT nomenclature; please see “Results”). To generate reconstructions of each of these NIEP portal subtypes, we performed 3D refinement with local orientation search on the best representative classes. This resulted in three final reconstructions of the NC1, NC2, and NC2-inv portal vertex subparticles with C5 symmetry, estimated at 4.26-, 4.29-, and 4.01-Å resolution using the 0.143 FSC criterion [figs. S3 (C to E and G) and S4 and table S1]. Of note, we took VC2 to be representative of NC2 in our structure analyses, as NC2 PVAT, while similar in general appearance to VC2 PVAT, had a higher degree of smearing likely due to its lack of localization via interaction with genome terminus (because NC2 is a NIEP configuration) [figs. S3 (D and H) and S4].

### Global reconstructions of portal vertex-resolved capsid

To visualize global capsid-scale features (i.e., filamentous PVAT densities and portal-referenced PVAT distribution), we used the determined orientations of each configuration (i.e., VC1, VC2, NC1, and NC2-inv) of portal vertex subparticles to generate their respective portal vertex-resolved, global capsid reconstructions. A total of 30,047 virion particles, correlating to a subset of VC1 portal vertex subparticles, were used to generate a VC1 global capsid reconstruction with C5 symmetry using 3D refinement with local orientation search of entire virus particle images. In similar fashion, VC2, NC1, and NC2-inv global capsid reconstructions were generated from corresponding subsets of 60,912, 9,742, and 8,424 virus particles, respectively. We did not pursue a global reconstruction of NC2, given its heterogeneity and similarity to VC2 (see previous section). Owing to the large computational requirement for refinement of entire virus particles (up to 1280 × 1280 pixels for a single virus particle), we performed global capsid refinement using 4×-binned particles for virions and 2×-binned particles for NIEPs. We thus achieved Nyquist resolution for all four global reconstructions of VC1, VC2, NC1, and NC2-inv with C5 symmetry at 10.88-, 10.88-, 6.44-, and 6.44-Å resolution, respectively (fig. S5 and table S1).

### C1 reconstruction of CVSC-bound penton vertex

The previous 3D classification we performed to extract unique portal vertex subparticles from the icosahedral capsid also resulted in 3D classes containing penton vertex subparticles with C5 symmetry. From these, we selected a class containing 374,732 penton vertex subparticles and performed symmetry expansion with relion_particle_symmetry_expand. This yielded five uniquely oriented vertices for every single penton vertex subparticle, for a total of 1,873,660 symmetry-expanded subparticles. We next generated a mask encompassing one CVSC-binding-register (situated above Ta and Tc triplexes; five possible registers surround a penton vertex) and surrounding capsid and performed focused 3D classification on the masked volume without imposed symmetry or orientation search. Of the four resulting classes, three classes exhibited a canonical Ta triplex register with three bound pp150 molecules, whereas one class exhibited clearly visible CVSC density in the masked CVSC-binding-register (Fig. 7A). We used all 355,778 CVSC-containing subparticles from this class for subsequent 3D refinement with local orientation search. This resulted in the C1 reconstruction of a CVSC-bound penton vertex with one CVSC-bound CVSC-binding-register estimated at 3.02-Å resolution at the 0.143 FSC criterion [figs. S3 (F and G) and S4 and table S1].

### Atomic modeling and structure interpretation

Reconstructed cryo-ET tomograms were visualized and interpreted using 3dmod in IMOD (*84*) and UCSF ChimeraX 1.6 (*89*). Tomogram segmentation as well as the calculation and placement of 3D markers corresponding to portal, capsid, and bulky tegument centroids used to determine PCT angles were performed manually and with the aid of virtual reality in ChimeraX.

Our cryo-EM reconstructions of PVAT-bound portal and penton vertices covered a range of features from rigid, high-resolution structures of capsid and capsid-proximal regions of PVAT to loosely bound, flexible capsid-distal PVAT features lower in resolution. To facilitate density map interpretation and model building efforts, for each reconstruction, we generated series of density maps with multiple levels of B-factor sharpening and Gaussian filtering using RELION (*88*) and ChimeraX to emphasize different structural features. We also made use of cryo-EM map improvement tools from the Phenix tool suite (*90*), including resolve_cryo_em, in our generation of local optimized maps.

Ab initio atomic models were built by assigning amino acid residues to the density map in Coot 0.9.8.1 (*91*) and with the aid of sequence-derived secondary structure prediction from Phyre2 (*92*). Briefly, Cα-backbone mainchains were generated using Coot's baton_build utility, and sequence-registered mainchains were manually refined using Coot’s regularize_zone and real_space_refine_zone tools. Completed models were then subjected to multiple iterations of automated refinement using real_space_refine in Phenix followed by manual adjustment of problematic residues in Coot to improve model geometry and map fit (table S1).

Refined models and molecular details were visualized, interpreted, and rendered for figure and movie display using ChimeraX. Local resolution maps of reconstructions were generated using ResMap (*93*).

### Application of AlphaFold2 in low-resolution density interpretation

AlphaFold2 (*61*) proved instrumental in our structure interpretation efforts in two modalities. The first was as a model building aid. In regions of suboptimal density map quality where native mainchain tracing and sequence registration were difficult, we generated AlphaFold2-predicted structures of the entire protein or local domain of interest using San Diego Supercomputer Center’s COSMIC^2^ web server (*94*). Predicted structures were then evaluated for accuracy using our experimental map data and, where applicable, used to guide mainchain tracing of novel proteins. Second, we used AlphaFold2 to generate predicted structures of pUL47 and pUL48 coiled-coil helices, which were not visible in their entirety in our focused subparticle reconstructions of PVAT but were visible in global capsid reconstructions. Bundles of predicted coiled-coil helices were then manually fit into our reconstruction using rigid-body docking tools in Coot (*91*) and ChimeraX (*89*).
