# Supplementary material for: The incredible bulk: Human cytomegalovirus tegument architectures uncovered by AI-empowered cryo-EM
Source: Sci Adv. 2024 Feb 23;10(8):eadj1640. doi: 10.1126/sciadv.adj1640 (PMC10889378; doi:10.1126/sciadv.adj1640)
Supplement: Supplementary file 1 — Figs. S1 to S8 Tables S1 and S2 Legends for movies S1 to S4 [file sciadv.adj1640_sm.pdf]

Science Advances
AAAS

Supplementary Materials for

The incredible bulk: Human cytomegalovirus tegument architectures uncovered by AI-empowered cryo-EM

Jonathan Jih *et al.*

Corresponding author: Z. Hong Zhou, hong.zhou@ucla.edu

Sci. Adv. **10**, eadj1640 (2024)
DOI: 10.1126/sciadv.adj1640

The PDF file includes:

Figs. S1 to S8
Tables S1 and S2
Legends for movies S1 to S4

Other Supplementary Material for this manuscript includes the following:

Movies S1 to S4

Fig. S1.

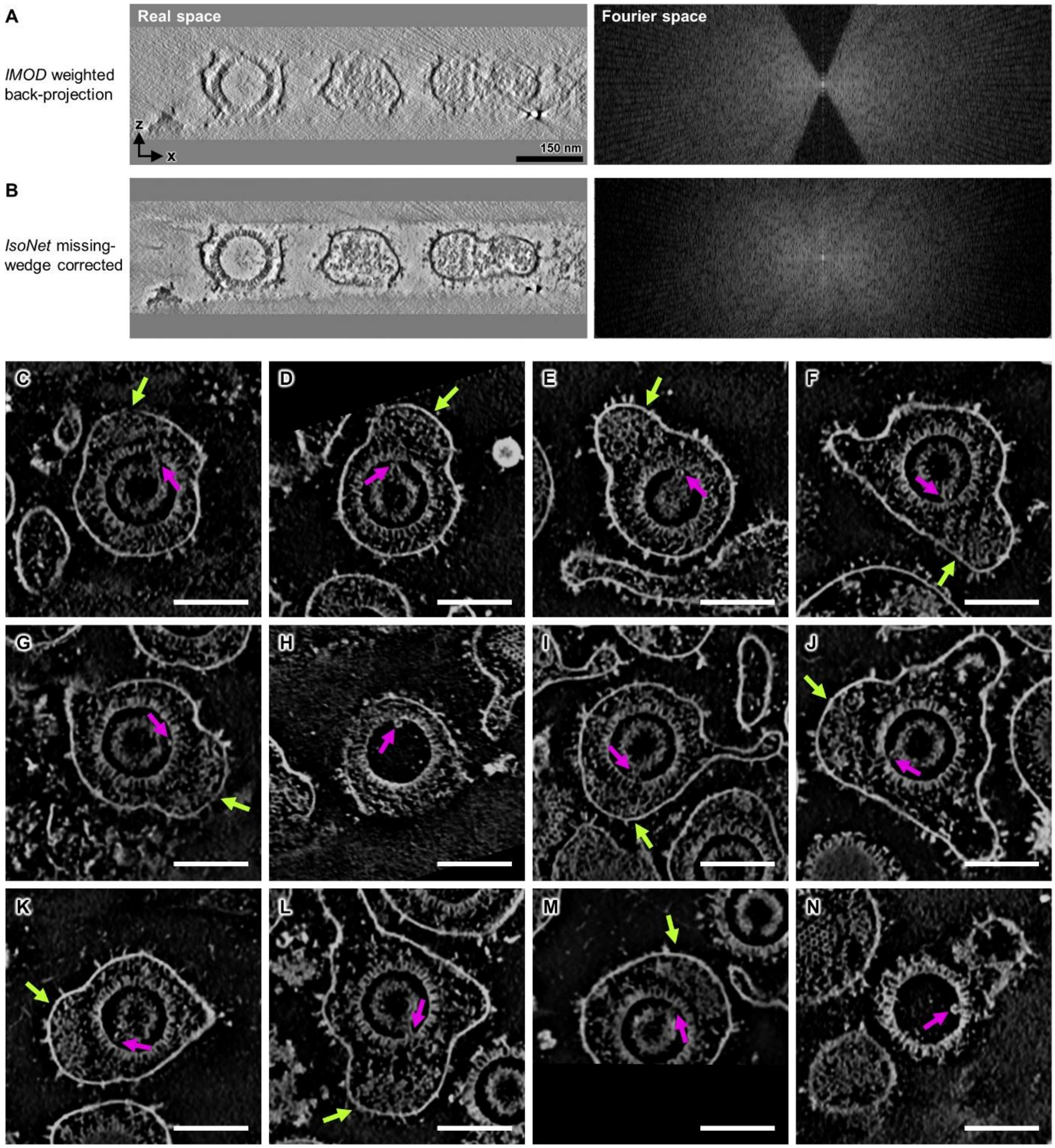

Fig. S1. Missing-wedge–corrected cryoET of HCMV enveloped particles. (**A**,**B**) Comparison of tomogram from Figure 1 before (A) and after (B) *IsoNet* (*49*) neural network training and correction. Real space features such as resolution of individual capsomers in the x-z plane are improved (left panels) and missing-wedge information in Fourier space (right panels) is recovered. (**C-N**) Tomogram slices showing NIEPs, both scaffold-containing and empty, with visible portal complex (purple arrows) and asymmetric tegument compartment (green arrows). Scale bar 100 nm.

Fig. S2.

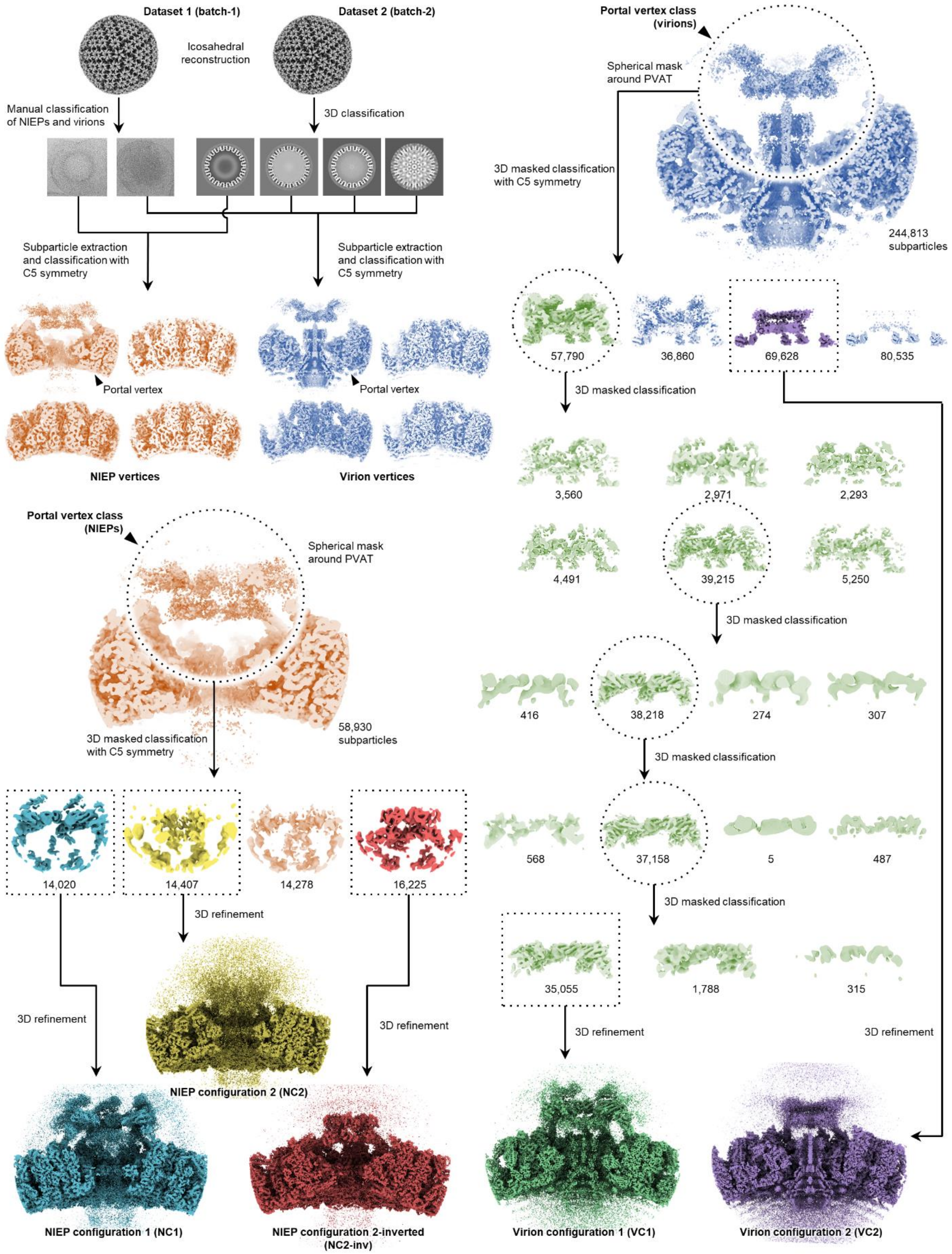

Dataset 1 (batch-1)
Dataset 2 (batch-2)
Icosahedral reconstruction
Manual classification of NIEPs and virions
3D classification
Subparticle extraction and classification with C5 symmetry
Subparticle extraction and classification with C5 symmetry
Portal vertex
Portal vertex
NIEP vertices
Virion vertices
Portal vertex class (NIEPs)
Spherical mask around PVAT
3D masked classification with C5 symmetry
58,930 subparticles
14,020
14,407
14,278
16,225
3D refinement
3D refinement
3D refinement
NIEP configuration 2 (NC2)
NIEP configuration 1 (NC1)
NIEP configuration 2-inverted (NC2-inv)
Portal vertex class (virions)
Spherical mask around PVAT
3D masked classification with C5 symmetry
244,813 subparticles
57,790
36,860
69,628
80,535
3D masked classification
3,560
2,971
2,293
4,491
39,215
5,250
3D masked classification
416
38,218
274
307
3D masked classification
568
37,158
5
487
3D masked classification
35,055
1,788
315
3D refinement
3D refinement
Virion configuration 1 (VC1)
Virion configuration 2 (VC2)

Fig. S2. Sequential classification and cryoEM subparticle reconstruction of portal vertex configurations. Flowchart illustrates the application of symmetry-relaxed sequential localized classification to extract the unique portal vertex and resolve PVAT configurations in NIEPs and virions.

Fig. S3.

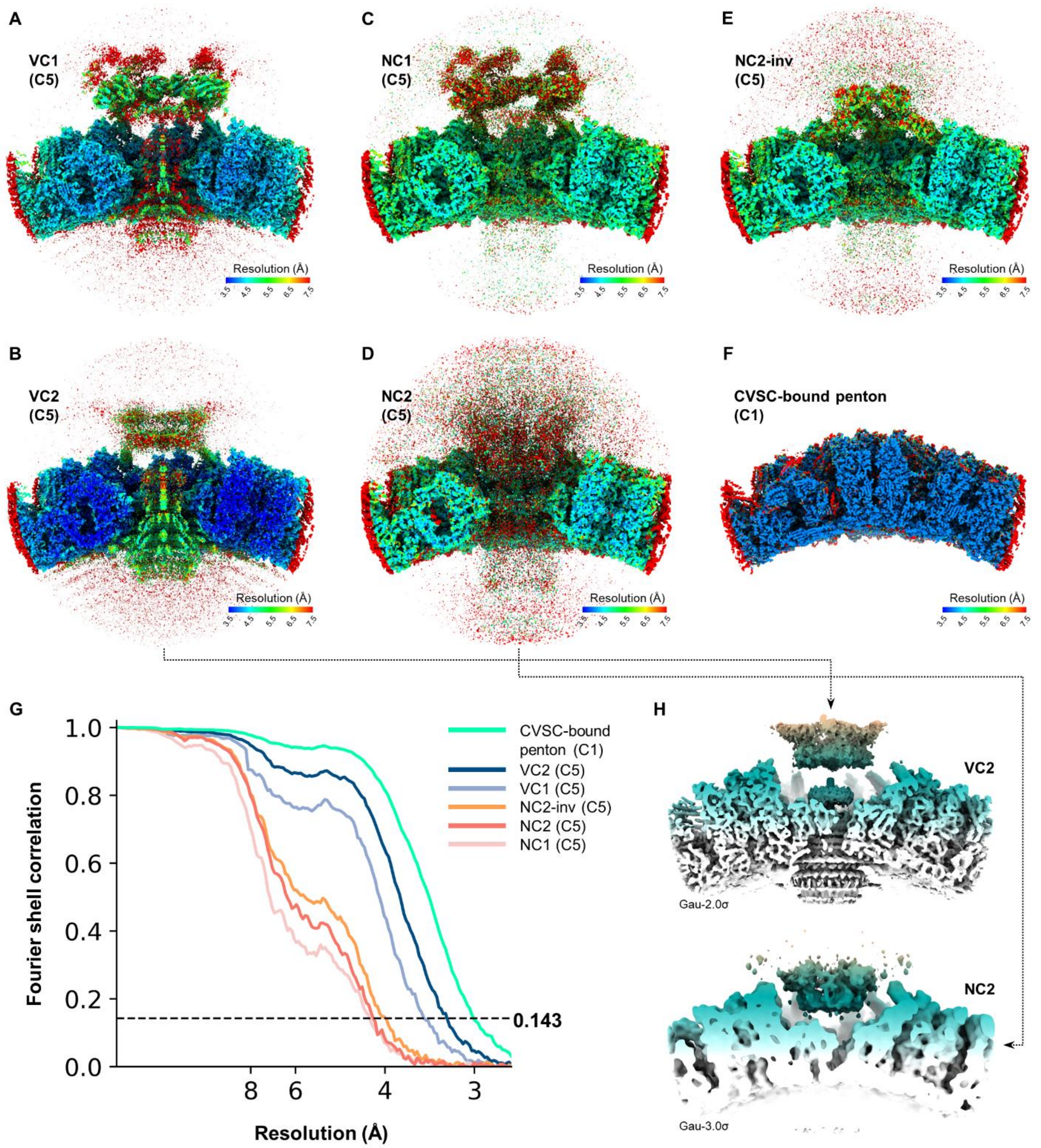

Fig. S3. Resolution assessment of cryoEM subparticle reconstructions. (**A**-**F**) Subparticle density maps of PVAT-decorated portal vertices (A-E) and CVSC-bound penton vertex (F) colored by local resolution estimated using *ResMap* (*93*). (**G**) Gold-standard FSC curves for all subparticle reconstructions, with the 0.143 criterion denoted by dashed line. (**H**) Comparison of VC2 and NC2 portal vertex reconstructions reveal similarities in PVAT morphology, despite NC2 PVAT's greater degree of disorder.

Fig. S4.

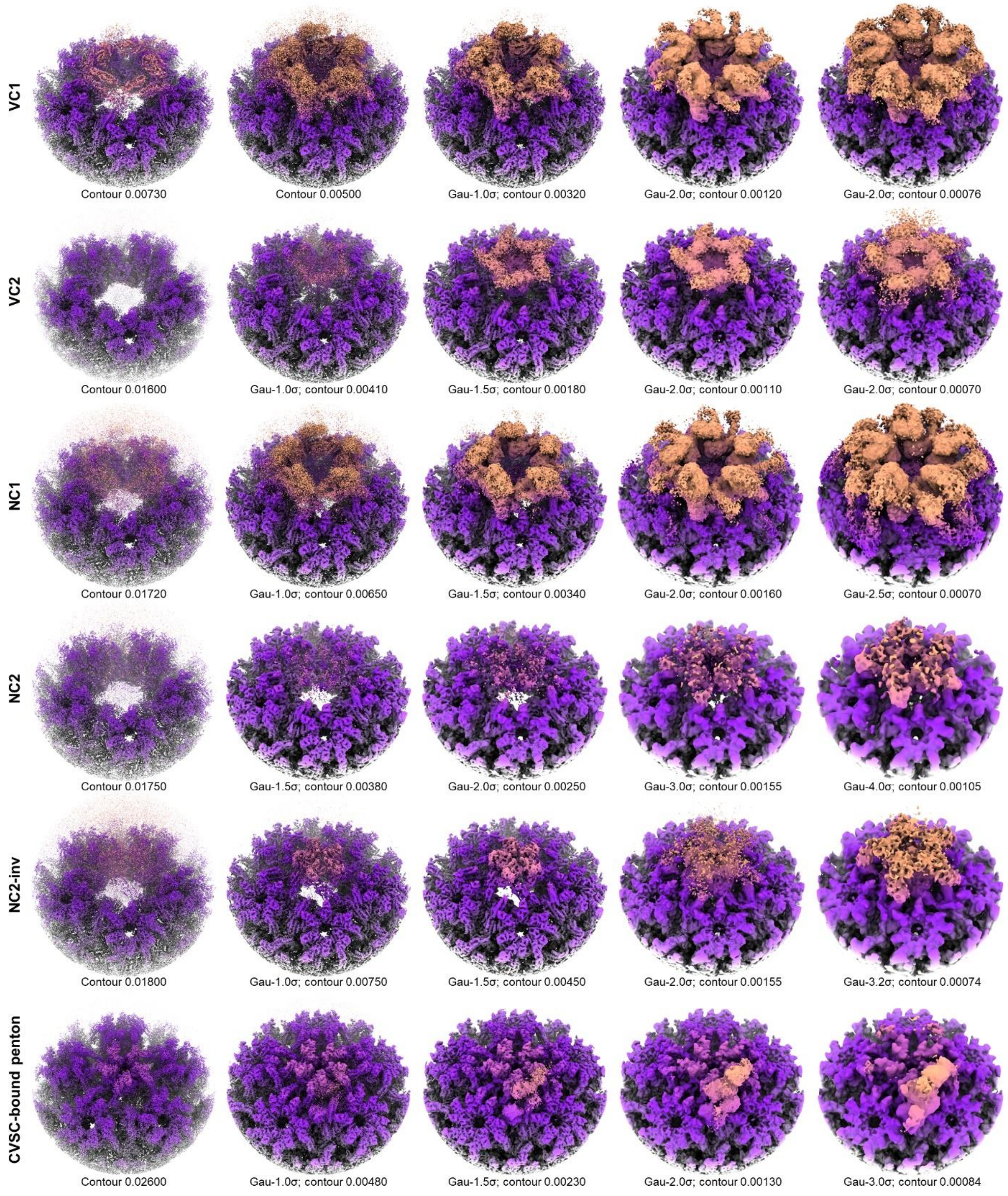

Fig. S4. Density series of cryoEM subparticle reconstructions. Density maps of PVAT-decorated portal vertices and CVSC-bound penton vertex displayed at increasing levels of Gaussian filtering and decreasing contour levels, highlighting the dynamic range of structural information available in our subparticle reconstructions.

Fig. S5.

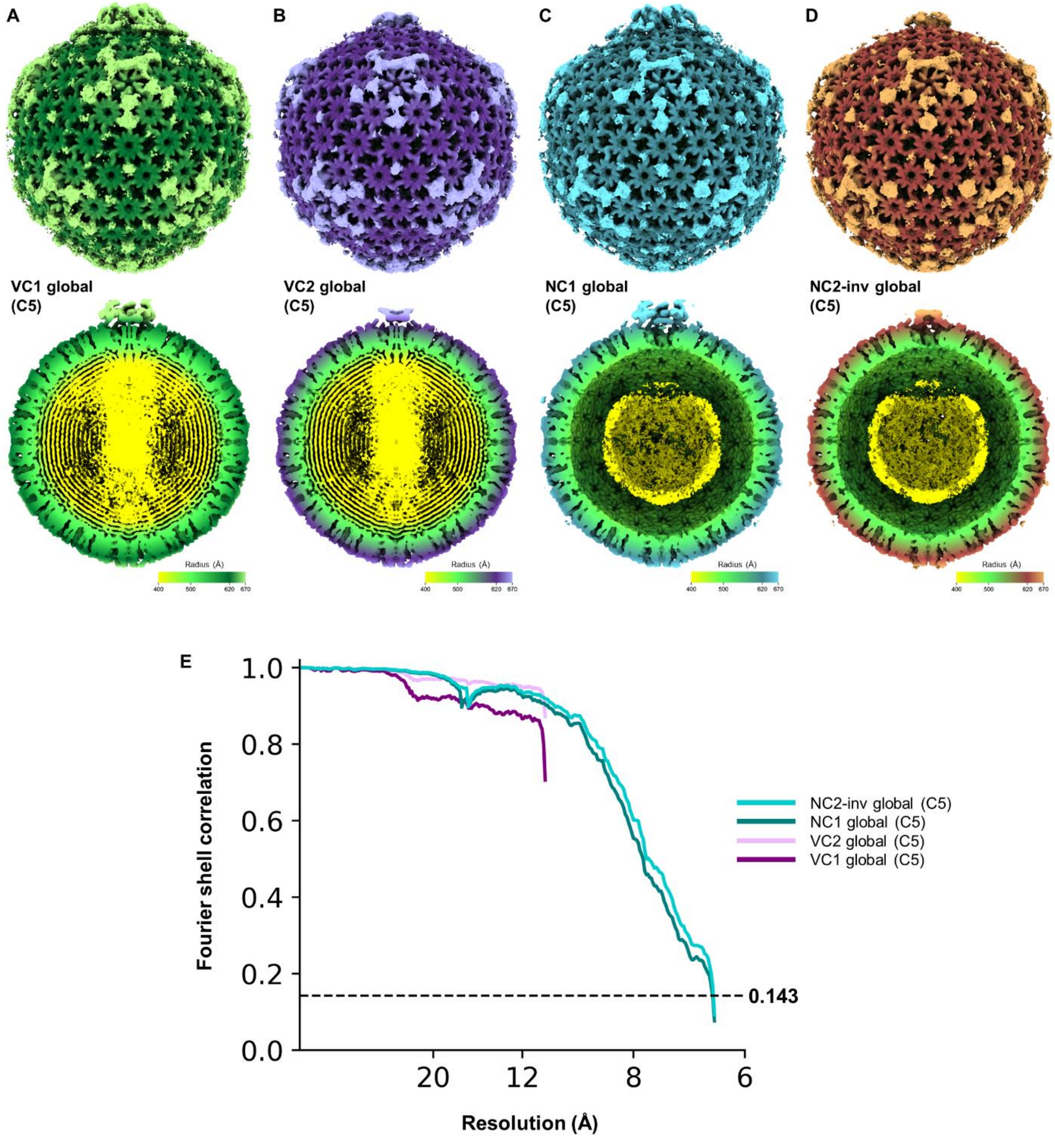

Fig. S5. Global cryoEM reconstructions of portal vertex-resolved capsids. (**A**-**D**) Radially-colored global reconstructions of portal vertex-resolved capsids show conserved and distinctive portal-biased tegumentation at penton vertices across all PVAT capsid types. Clipped-views show concentric shells of genomic density in virions (A,B) and scaffold core in NIEPs (C,D). (**E**) Gold-standard FSC curves for all global capsid reconstructions by PVAT type, all of which reached Nyquist resolution.

Fig. S6.

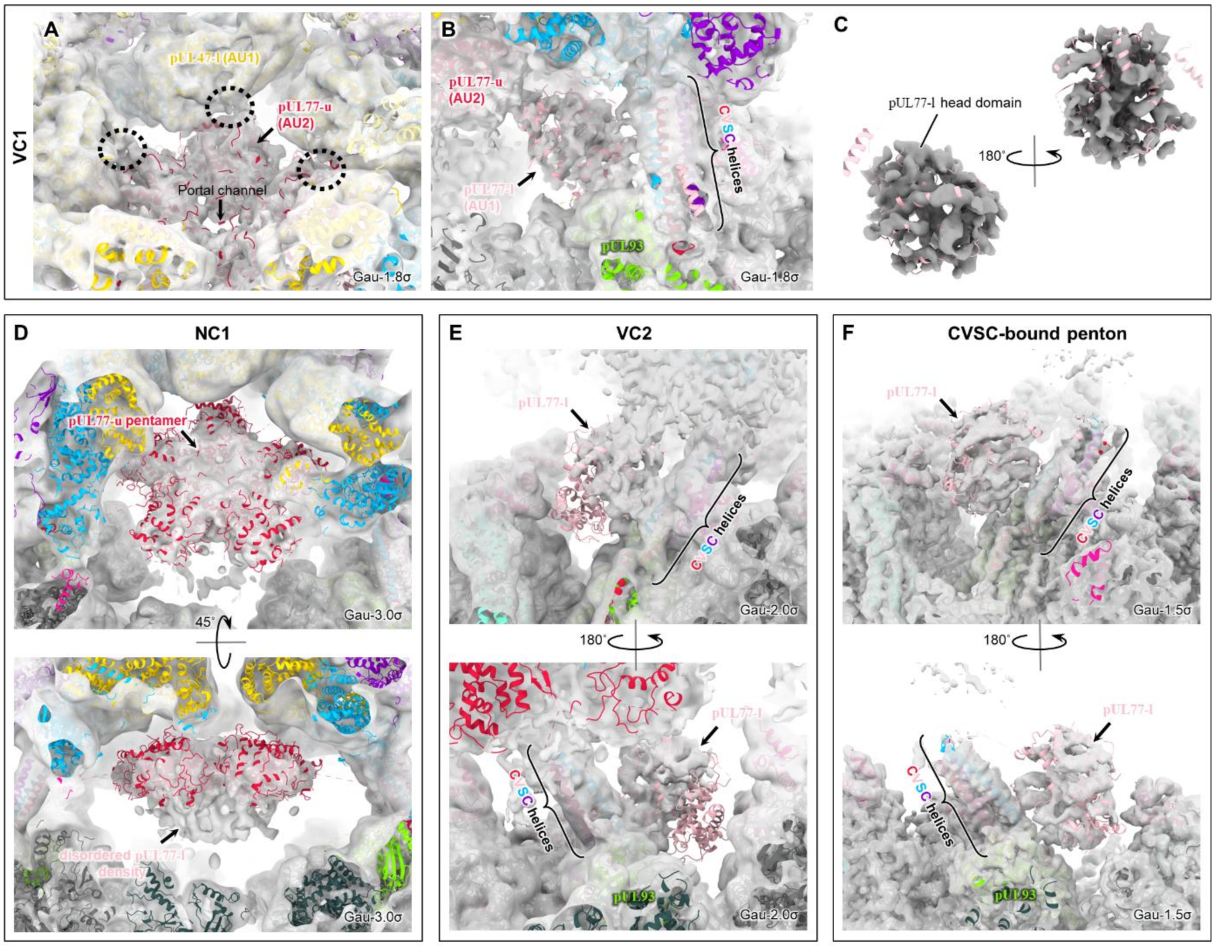

Fig. S6. pUL77 configurations and loosely bound states. (**A**) Density contact visible under Gaussian filtering (dashed circles) occurs between VC1 pUL77-u and pUL47-l through unstructured (and unmodeled) loops. (**B**,**C**) Close-up views of model-to-map fit of pUL77-l in VC1 portal vertex reconstruction. The pUL77-l model was generated by rigid-body fitting pUL77-u into pUL77-l density. (**D**) NC1 portal vertex reconstruction shows similar pUL77-u pentamerization as in VC1 (NC1 models rigid-body fit from VC1) but with disordered pUL77-l density beneath the pentamer, correlating with the lack of genome terminus in the NC1 portal channel. (**E**,**F**) Reconstructions of VC2 portal vertex (E) and CVSC-bound penton vertex (F) reveal loosely bound and/or low-occupancy copies of pUL77-l adjacent to CVSC helices, in a position analogous to that of density first observed in KSHV and postulated to be pORF19, KSHV’s pUL77 homolog (*43*).

Fig. S7.

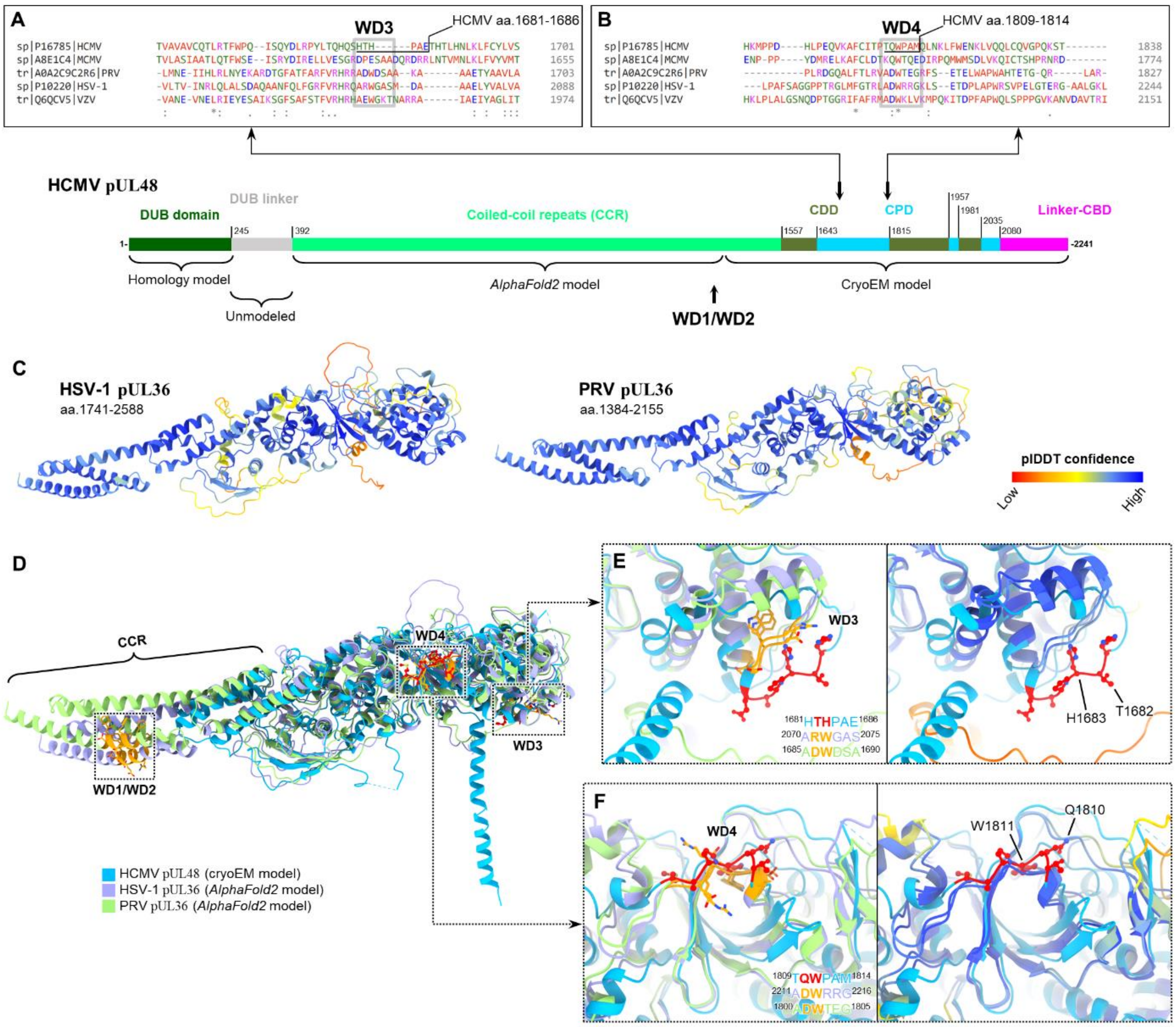

Fig. S7. Kinesin-binding motifs in herpesvirus large tegument protein homologs. (**A**,**B**) Schematic depicting protein sequence alignment of WD3 (A) and WD4 (B) putative kinesin-binding motifs (*24*) in the large tegument proteins of three alphaherpesviruses (PRV, HSV-1, and VZV) and two betaherpesviruses (HCMV and MCMV) and their presumed location within HCMV pUL48. (**C**) *AlphaFold2*-predicted models of the large tegument proteins of HSV-1 (pUL36) and PRV (pUL36), colored by plDDT confidence score. (**D**) HCMV pUL48 atomic model superposed with HSV-1 and PRV pUL36 *AlphaFold2* models. Dashed boxes correspond to known regions of kinesin interaction. (**E**,**F**) Structural alignment of WD3 (E) and WD4 (F) indicates possible conservation of the WD4 motif in HCMV pUL48.

Fig. S8.

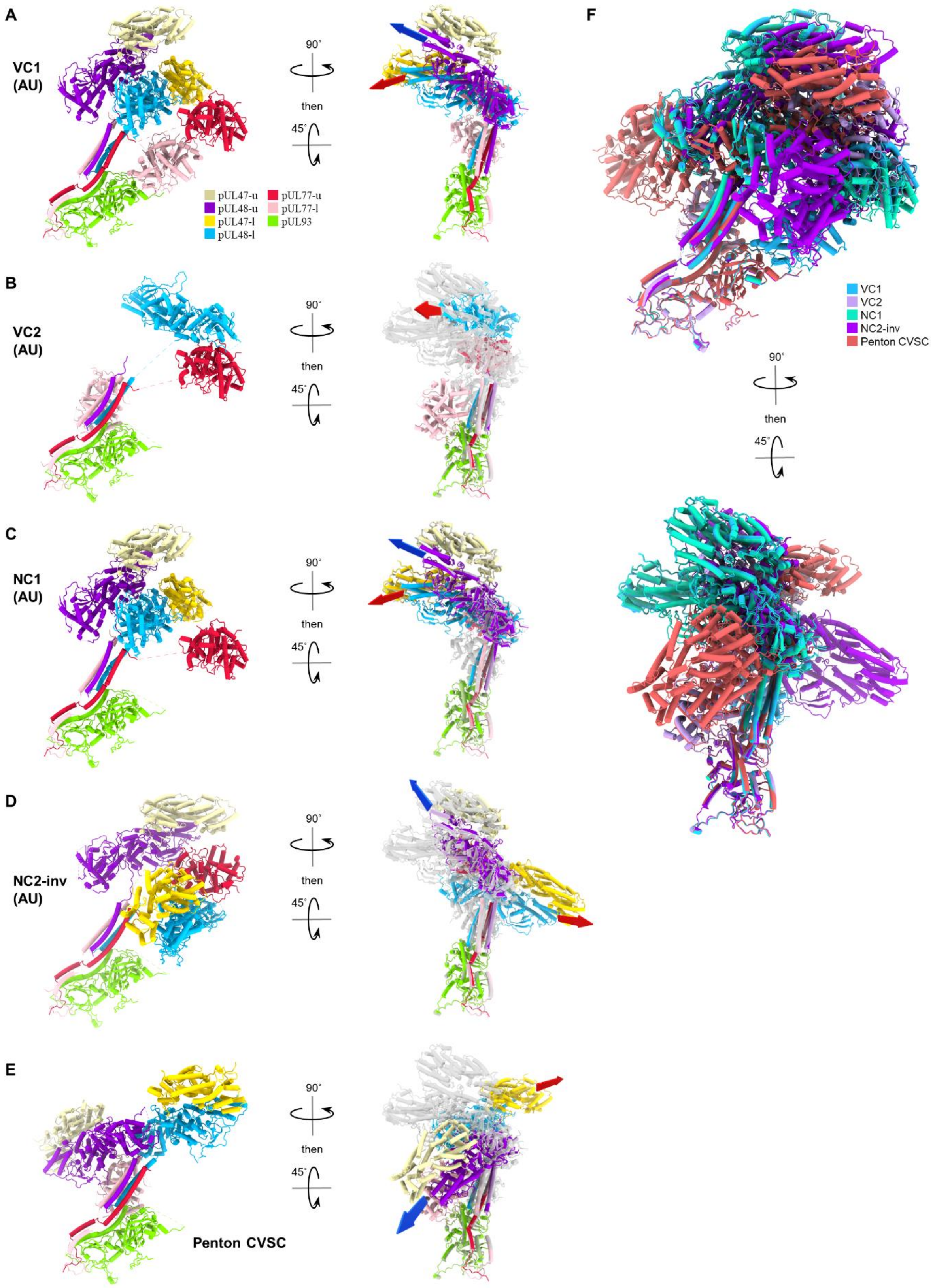
A
VC1
(AU)
pUL47-u
pUL48-u
pUL47-l
pUL48-l
pUL77-u
pUL77-l
pUL93
90°
then
45°
B
VC2
(AU)
C
NC1
(AU)
D
NC2-inv
(AU)
E
Penton CVSC
F
VC1
VC2
NC1
NC2-inv
Penton CVSC

Fig. S8. Structural permutations of vertex-associated tegument components. (**A**-**E**) Left panels show the full asymmetric units of PVAT (A-D) and penton CVSC (E), including loosely bound and/or low-occupancy components. Red and blue arrows in right panels indicate the general direction of pUL48 coiled-coil repeats projecting from each pUL47/48 dimer. Right panels in (B-E) also depict the VC1 asymmetric unit in gray for structural comparison. (**F**) Superposition of all PVAT and penton CVSC asymmetric units, aligned with respect to pUL93.

Table S1. CryoEM data collection, refinement, and modeling statistics. Asterisks (*) denote categories where two reported values correspond to batch-1 and batch-2 collected data (i.e., batch-1/batch-2).

Table S1. CryoEM data collection, refinement, and modeling statistics

	VC1 portal vertex (EMD-41194, PDB 8TEP)	VC2 portal vertex (EMD-41200, PDB 8TES)	NC1 portal vertex (EMD-41201, PDB 8TET)	NC2 portal vertex (EMD-41206)	NC2-inv portal vertex (EMD-41202, PDB 8TEU)	CVSC-bound penton vertex (EMD-41204, PDB 8TEW)	VC1 global capsid (EMD-41213)	VC2 global capsid (EMD-41214)	NC1 global capsid (EMD-41215)	NC2-inv global capsid (EMD-41216)
**Data collection and processing**										
Magnification*	31,120x/ 105,000x	31,120x/ 105,000x	31,120x/ 105,000x	31,120x/ 105,000x	31,120x/ 105,000x	31,120x/ 105,000x	31,120x/ 105,000x	31,120x/ 105,000x	31,120x/ 105,000x	31,120x/ 105,000x
Voltage (kV)	300	300	300	300	300	300	300	300	300	300
Electron exposure ($e^-/Å^2$)*	37.8/47.2	37.8/47.2	37.8/47.2	37.8/47.2	37.8/47.2	37.8/47.2	37.8/47.2	37.8/47.2	37.8/47.2	37.8/47.2
Defocus range (μm)	-1.0 to -3.0	-1.0 to -3.0	-1.0 to -3.0	-1.0 to -3.0	-1.0 to -3.0	-1.0 to -3.0	-1.0 to -3.0	-1.0 to -3.0	-1.0 to -3.0	-1.0 to -3.0
Pixel size (Å)	1.36	1.36	1.36	1.36	1.36	1.36	5.44	5.44	3.22	3.22
Symmetry imposed	C5	C5	C5	C5	C5	C1	C5	C5	C5	C5
Initial particle images (no.)	244,813	244,813	58,930	58,930	58,930	355,778	30,047	60,912	9,742	8,424
Final particle images (no.)	35,055	69,628	14,020	14,407	16,225	355,778	30,047	60,912	9,742	8,424
Map resolution (Å)	3.50	3.27	4.26	4.29	4.01	3.02	10.88	10.88	6.44	6.44
FSC threshold	0.143	0.143	0.143	0.143	0.143	0.143	0.143	0.143	0.143	0.143
Estimated resolution range (Å)	3.5-7.5	3.3-7.5	4.3-7.5	4.3-7.5	4.0-7.5	3.0-7.5	--	--	--	--
Map sharpening B factor ($Å^2$)	-140.2	-139.8	-201.2	-199.2	-188.7	-96.5	0	0	0	0
**Model refinement**										
Model-to-map fit, mask CC	0.84	0.81	0.70	--	0.66	0.91	--	--	--	--
Model composition										
Chains	26	24	24		24	27				
Non-hydrogen atoms	112,987	88,005	87,998		96,759	104,261				
Protein residues	14,141	11,051	11,050		12,139	13,080				
Bond statistics, RMSD										
Bond lengths (Å)	0.005	0.007	0.005		0.004	0.005				
Bond angles (°)	1.045	1.108	1.036		0.986	0.993				
Validation										
MolProbity score	1.70	1.59	1.78		1.67	1.46				
Clash score	7.69	5.24	7.79		6.79	4.03				
Rotamer outliers (%)	0.07	0.22	0.01		0.11	0.11				
Ramachandran plot										
Outliers (%)	0.09	0.05	0.09		0.12	0.09				
Allowed (%)	3.93	4.40	5.09		4.12	3.83				
Favored (%)	95.99	95.54	94.82		95.76	96.08				
Rama Z-score, RMSD										
Whole	-0.11	-0.63	-0.86		-0.42	0.48				
Helix	0.67	-0.20	-0.10		0.31	0.98				
Sheet	-0.07	0.28	-0.05		0.05	0.52				
Loop	-0.68	-0.73	-1.07		-0.82	-0.24				

Table S2. Summary of key materials and resources

	Source	*Identifier*
Viruses and cell lines		
Human herpesvirus 5 strain AD169	ATCC	VR-538
Human fibroblast MRC-5 cells	ATCC	CCL-171
Chemical reagents and media		
Dulbecco's Modified Eagle Medium (DMEM)	ATCC	30-2002
Eagle's Minimum Essential Medium (EMEM)	ATCC	30-2003
Fetal bovine serum – premium, heat inactivated	R&D Systems	Cat#S11150H
Gibco phosphate buffered saline, pH 7.4	ThermoFisher Scientific	Cat#10-010-023
NP-40	ThermoFisher Scientific	Cat#85124
Electron microscopy-related materials		
Quantifoil Holey Carbon Grids (2/1)	Quantifoil	https://www.quantifoil.com/products/quantifoil/quantifoil-circular-holes
FEI Vitrobot Mark IV	ThermoFisher Scientific	https://assets.thermofisher.com/TFS-Assets/MSD/Datasheets/Thermo-Scientific-Vitrobot-Datasheet.pdf
ThermoFisher Titan Krios Cryo-TEM	ThermoFisher Scientific	https://www.thermofisher.com/us/en/home/electron-microscopy/products/transmission-electron-microscopes.html
Gatan Quantum Imaging Filter	Gatan	https://www.gatan.com/products/tem-stem-products
Gatan K2 Summit direct electron detection camera	Gatan	https://www.gatan.com/products/tem-stem-products
Volta Phase Plate	ThermoFisher Scientific	https://www.thermofisher.com/us/en/home/electron-microscopy/products/transmission-electron-microscopes.html
Data processing and visualization software		
SerialEM 3.6	(*82*)	https://bio3d.colorado.edu/SerialEM/

Leginon 3.3	(*85*)	https://emg.nysbc.org/redmine/projects/leginon/wiki/Leginon_Homepage
MotionCor2	(*83*)	https://emcore.ucsf.edu/ucsf-software
IMOD 4.11	(*84*)	https://bio3d.colorado.edu/imod/
IsoNet	(*49*)	https://isonetcryoet.com/
CTFFIND3	(*86*)	https://grigoriefflab.umassmed.edu/ctf_estimation_ctffind_ctftilt
Topaz v0.2	(*87*)	https://emgweb.nysbc.org/topaz.html
RELION 3.1	(*88*)	https://github.com/3dem/relion
Capsid vertex subparticle reconstruction scripts	(*42*)	https://github.com/procyontao/Herpesportal
Coot 0.9.8.1	(*91*)	https://www2.mrc-lmb.cam.ac.uk/personal/pemsley/coot/source/releases/
UCSF ChimeraX 1.6	(*89*)	https://www.cgl.ucsf.edu/chimerax/
AlphaFold2	(*61*)	https://github.com/deepmind/alphafold
Phenix dev-4788	(*90*)	https://phenix-online.org/
ResMap	(*93*)	https://resmap.sourceforge.net/

Movie S1.

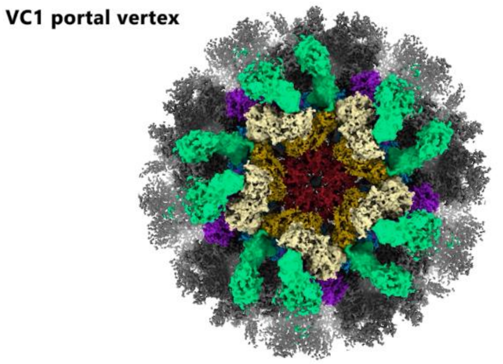

[movie still]

Movie S1. Structures of the VC1 portal vertex. Subparticle reconstruction and models of VC1 PVAT-decorated virion portal vertex. Composite map shown with filtering and contours chosen to best display overall structural features.

Movie S2.

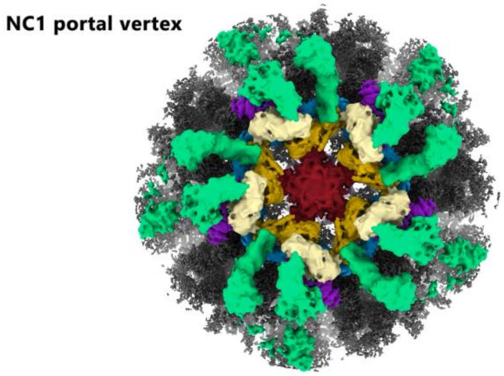

[movie still]

Movie S2. Structures of the NC1 portal vertex. Subparticle reconstruction and models of NC1 PVAT-decorated NIEP portal vertex. Composite map shown with filtering and contours chosen to best display overall structural features.

Movie S3.

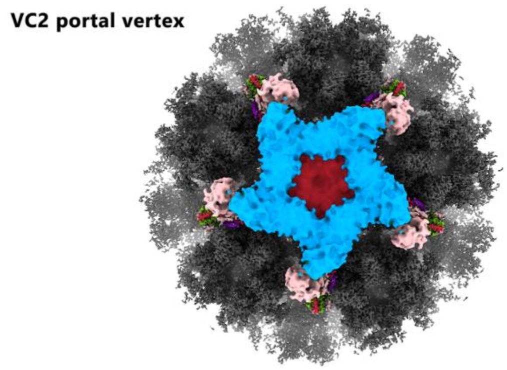

[movie still]

Movie S3. Structures of the VC2 portal vertex. Subparticle reconstruction and models of VC2 PVAT-decorated virion portal vertex. Composite map shown with filtering and contours chosen to best display overall structural features.

Movie S4.

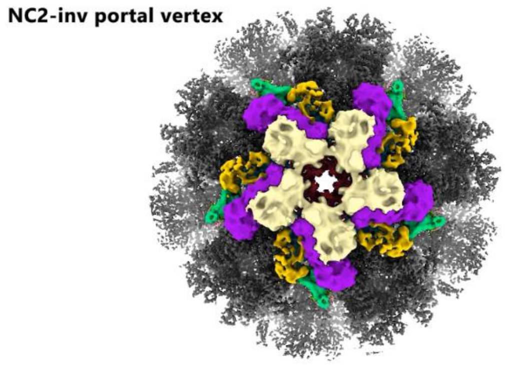

[movie still]

Movie S4. Structures of the NC2-inv portal vertex. Subparticle reconstruction and models of NC2-inv PVAT-decorated NIEP portal vertex. Composite map shown with filtering and contours chosen to best display overall structural features.
